# The effects of n-6 polyunsaturated fatty acid deprivation on the inflammatory gene response to lipopolysaccharide in the mouse hippocampus

**DOI:** 10.1186/s12974-019-1615-0

**Published:** 2019-11-27

**Authors:** Shoug M. Alashmali, Lin Lin, Marc-Olivier Trépanier, Giulia Cisbani, Richard P. Bazinet

**Affiliations:** 1 Department of Clinical Nutrition, Faculty of Applied Medical Sciences, King Abdul Aziz University, Jeddah, Saudi Arabia; 2 Department of Nutritional Sciences, Faculty of Medicine, University of Toronto, Toronto, ON Canada

**Keywords:** Neuroinflammation, Lipopolysaccharide, N-6 polyunsaturated fatty acids, mRNA, Hippocampus, Arachidonic acid, Linoleic acid

## Abstract

**Background:**

Neuroinflammation is thought to contribute to psychiatric and neurological disorders such as major depression and Alzheimer’s disease (AD). N-6 polyunsaturated fatty acids (PUFA) and molecules derived from them, including linoleic acid- and arachidonic acid-derived lipid mediators, are known to have pro-inflammatory properties in the periphery; however, this has yet to be tested in the brain. Lowering the consumption of n-6 PUFA is associated with a decreased risk of depression and AD in human observational studies. The purpose of this study was to investigate the inflammation-modulating effects of lowering dietary n-6 PUFA in the mouse hippocampus.

**Methods:**

C57BL/6 male mice were fed either an n-6 PUFA deprived (2% of total fatty acids) or an n-6 PUFA adequate (23% of total fatty acids) diet from weaning to 12 weeks of age. Animals then underwent intracerebroventricular surgery, in which lipopolysaccharide (LPS) was injected into the left lateral ventricle of the brain. Hippocampi were collected at baseline and following LPS administration (1, 3, 7, and 14 days). A microarray (*n* = 3 per group) was used to identify candidate genes and results were validated by real-time PCR in a separate cohort of animals (*n* = 5–8 per group).

**Results:**

Mice administered with LPS had significantly increased Gene Ontology categories associated with inflammation and immune responses. These effects were independent of changes in gene expression in any diet group. Results were validated for the effect of LPS treatment on astrocyte, cytokine, and chemokine markers, as well as some results of the diets on *Ifrd2* and *Mfsd2a* expression.

**Conclusions:**

LPS administration increases pro-inflammatory and lipid-metabolizing gene expression in the mouse hippocampus. An n-6 PUFA deprived diet modulated inflammatory gene expression by both increasing and decreasing inflammatory gene expression, without impairing the resolution of neuroinflammation following LPS administration.

## Background

Neuroinflammation is an active defensive process characterized by many reactions including the release of arachidonic acid (ARA; n-6 PUFA), and ARA-derived pro-inflammatory lipid mediators, which in turn regulate inflammatory genes. Neuroinflammation is increasingly recognized as an important component, either causal or as a secondary response, of neurodegenerative diseases, including Alzheimer’s disease (AD) and Parkinson’s disease, as well as psychiatric disorders, such as major depression [1–3].

The brain is an immune-privileged tissue compared to the peripheral immune system, containing its typical prominent immune cells: microglia and astrocytes [4, 5]. Microglia and astrocyte activation, pro-inflammatory cytokines such as interleukin *(Il)-1β*, *Il-6*, and tumor necrosis factor (*Tnf*), chemokines such as *Ccl5*, or other neuroinflammatory markers, as well as reactive oxygen species, are elevated in animal models of neuroinflammation [6–8] and in human subjects with neurological disorders [9–11].

In the brain, n-6 PUFA makes up approximately 10% of total fatty acids [12, 13]. ARA is the most abundant n-6 PUFA in the brain and is involved in many functions in the central nervous system, including neurotransmission, neurogenesis, and neuroinflammation [14, 15]. Altered n-6 PUFA metabolism has been linked to the pathogenesis of both neurological and psychiatric disorders [16–18]. Studies in humans have suggested a pro-inflammatory role of n-6 PUFA in many brain disorders, such as AD and depression [18, 19]. While the majority of n-6 PUFA have pro-inflammatory properties as precursors of prostaglandins and leukotrienes, some n-6 PUFA can also be converted to anti-inflammatory mediators including lipoxins and epoxyeicosatrienoic acids (EETs) [20–23]. Because n-6 PUFA-derived lipid mediators can act as both pro- and anti-inflammatory, it is unclear how dietary n-6 PUFA modulates neuroinflammation.

Despite the fact that animal studies generally demonstrate that lowering n-6 PUFA lowers brain ARA and ARA-derived lipid mediators [12, 13, 24] and/or enzymes of the ARA cascade [25, 26], little is known regarding their effects on brain inflammation and its resolution. Studies have evaluated only a few pro-inflammatory markers at one time point [26]. The goal of this study was to examine the hippocampal response to an intracerebroventricular (icv) injection of lipopolysaccharide (LPS) over 14 days in mice consuming either an n-6 PUFA deprived or adequate diet. The administration of icv LPS is known to induce neuroinflammation by activating the toll-like receptor 4 and promoting a strong innate immune response accompanied by the secretion of pro-inflammatory cytokines and chemokines. We identified some changes in inflammation associated gene expression with dietary n-6 PUFA manipulation; however, no impairment in the resolution response to LPS was observed.

## Methods

### Animals and diets

The present experiment was carried out in accordance with the guidelines of the Canadian Council on Animal Care (protocol # 20011827). Mice were maintained under controlled light (14/10 light/dark cycle) and temperature conditions (21 °C) in the Department of Comparative Medicine animal facility at the University of Toronto, with ad libitum access to food and water.

C57BL/6 male mice were purchased from Charles River Laboratories (Saint-Constant, QC, Canada) and were received at the animal facility at 2 weeks of age with their dams. After a week of acclimatization, mice were randomly weaned onto either an n-6 PUFA 23% adequate or an n-6 PUFA 2% deprived diet (Dyets Inc., Bethlehem, PA, USA), in which 40% of the safflower oil in n-6 PUFA adequate diet was replaced with hydrogenated coconut oil. We used “adequate” and “deprived” as relative terms to maintain consistency with the previous literature [12, 13] to describe the 23% and 2% of total fatty acids LA levels, respectively, in the diets. The amount of linoleic acid in n-6 PUFA deprived diet is 10% of the minimum requirement of LA for rodents based on the AIN-93 standard diet (12 mg/g, 4% of energy) [27], but rodents do not show significant signs of LA deficiency. The level of LA in the n-6 PUFA adequate diet is comparable to the recommended level of LA for humans: 1000–1500 mg of linoleic acid per 100 g of diet, which provides ∼ 2–3% of energy [28, 29]. However, the amount of LA in the AIN-93 diet is based on the prevention of essential fatty acid deficiency for both linoleic acid and alpha-linolenic acid; therefore, the requirement of linoleic acid may have been overestimated [30].

The fatty acid compositions of the diets have been reported previously [13]. The main fatty acids in the n-6 PUFA adequate diet were linoleic (18:2n-6, 23.2%), oleic (18:1n-9, 7.5%), lauric (12:0, 26.2%), and palmitic (16:0, 8.8%). The most abundant fatty acids as a percent of total fatty acids in the n-6 PUFA deprived diet were linoleic (1.7%), oleic (6%), palmitic (9.5%), and lauric (12:0, 40.9%). Approximately 3.5% of total fatty acids were alpha-linolenic acid in both diets. Both ARA and docosahexaenoic acid (DHA) were not detected in either diet.

### Intracerebroventricular administration of LPS

At 12 weeks of age, 9 weeks after weaning, animals underwent icv surgery, in which LPS was injected into the left lateral ventricle of the brain as described previously [7]. LPS (*E*. *coli* stereotype 055:B5, Sigma Aldrich, St-Louis, MO, USA) was diluted to 1 μg in 1 μl of 0.9% of sterile saline. Briefly, mice were anesthetized, weighed, and immobilized in a stereotaxic setup with a digital reader (Stoelting, Wood Dale, IL, USA), and 150 μl of 0.03% sensorcaine was injected s.c. at the incision site. Following the incision and exposing the skull, a small hole was drilled − 0.1 mm, medial/lateral and − 0.5 mm anterior/posterior to the bregma. Five microliters of LPS was then injected at a depth of − 2.4 mm from the surface of the skull at a rate of 1 μl/min over 5 min by an electronic Stereotaxic Injector (Stoelting). The accuracy of the LPS injection to the left lateral ventricle was confirmed by periodic injection of Evan’s blue dye. Animal body weights were measured, after which mice were euthanized at 1, 3, 7, and 14 days following surgeries; as described below. These time points were selected based on our recent publication [7], where the time course of activated microglia and other inflammatory markers including cytokines and chemokines detected at 3 days and returned to baseline by 14 days, and are therefore time points where inflammation and resolution is likely to be detected. Non-surgery animals were used throughout the study as a reference control. In order to minimize the number of animals required, we did not perform sham surgeries as we have previously reported that icv LPS or amyloid beta induce a stronger inflammatory response as compared to sham surgery [8, 31].

### Brain collection for RNA measurements

For gene expression measurements, mice were euthanized by CO_2_ asphyxiation. Brains were rapidly harvested, and the left hippocampus (the ipsilateral side of LPS administration) was dissected and flash frozen with liquid nitrogen. Samples were stored at − 80 °C until further use.

### RNA extraction

Ipsilateral hippocampi from CO_2_ asphyxiated animals were homogenized in 150 μl Trizol (ThermoFisher Scientific, Waltham, MA, USA) with a Kimbel Kontes pestle homogenizer (Fisher Scientific, Waltham, MA, USA) as previously described [31]. Briefly, an additional 850 μl of Trizol was added to the samples. RNA was extracted according to the manufacturer’s instructions. RNA efficiency and the presence of contaminants were assessed with a Nanodrop 1000 Spectrophotometer (Nanodrop Technologies, Wilmington, DE, USA). The integrity of isolated RNA was measured in all microarray samples with BioAnalyzer Assay (Agilent 2100, Santa Clara, CA, USA); RNA integrity number values were higher than 8.

### Microarray analysis

A microarray (Affymetrix Gene ST arrays) was conducted to identify patterns of inflammatory gene expression associated with the LPS administration and diet. As previously described [31], extracted RNA was reverse transcribed with a WT Expression Kit (ThermoFisher), then single-stranded complementary DNA was fragmented and labeled according to the Affymetrix WT fragmentation and labelling protocol. cDNA was hybridized to the arrays with an Affymetrix Mouse Gene 2.0 ST GeneChip (ThermoFisher) for 18 h at 45 °C at 60 RPM to process it for analysis. Hybridization controls were similar across all arrays, indicating successful hybridization. Data was then imported into GeneSpring v13.1.1 (Agilent) for analysis. Data were normalized using a standard (for Affymetrix ST arrays) known as robust multi-array average (RMA) 16 normalization, followed by a median centred normalization per probe set. Data was filtered to remove probes with signals below the 20th percentile of the distribution of intensities for all samples. The final list contained 27,661 probe sets.

### Real time-qPCR

A group of genes driving categorical enrichment, by LPS administration or diet, from the microarray were used for validating the microarray results. Gene expression was measured in the same samples that were used for the microarray analysis as well as an independent cohort of CO_2_-asphyxiated animals to increase the sample size. Extracted RNA was reverse transcribed using a High Capacity cDNA Reverse Transcription Kit (ThermoFisher) according to the manufacturer’s instructions. Gene expression was measured using TaqMan gene expression assays (ThermoFisher) for lymphocyte antigen 6 complex, locus A (*Ly6A*, Mm04337234_mH), glial fibrillary acidic protein (*Gfap*, Mm01253033_m1) and the high affinity immunoglobulin gamma Fc region 1 (*Fcgr1*, Mm00438874_m1), chemokine (C-Cmotif) ligand 12 (*Ccl12*, Mm01617100_m1), interferon regulatory factor 7 (*Irf7*, Mm00516788_m1), phospholipase A_2_, group IVA (cytosolic, calcium-dependent) (*Pla2g4a*, Mm00447040_m1), chemokine (C-Cmotif) ligand 19 (*Ccl19*, Mm00839966_g1), interferon regulatory factor 2 (*Irf2*, Mm00515206_m1), interferon gamma receptor 2 (*Ifngr2*, Mm00492626_m1), major facilitator superfamily domain-containing protein 2 (*Mfsd2a*, Mm01192208_m1), *Ifrd2* (Mm00518083_m1), and TaqMan Gene Expression 2X Master Mix (ThermoFisher) as per manufacturer’s instructions. Each 10-μl reaction was run in triplicate in a 384-well optical plate on a 7900 HT Real-time PCR machine (Applied Biosystems, Foster City, CA, USA) with an initial incubation at 95 °C for 10 min, followed by 40 cycles of 95 °C for 15 s and 60 °C for 60 s as described previously [32]. Results are expressed as fold change from baseline non-surgery animals, calculated by using the equation 2^−ΔΔCt^ normalized to glyceraldehyde 3-phosphate dehydrogenase (*Gapdh*, Mm99999915_g1) and hypoxanthine-guanine phosphoribosyltransferase (*Hprt*, Mm03024075_m1). *Gapdh* and *Hprt* were selected as housekeeping genes based on their stability in the microarray study (Additional file 1; Figure S1) and their previous use in other papers measuring similar genes [33]. The stability of *Gapdh* and *Hprt* gene expressions across the LPS surgery and diet groups was confirmed in the full results of qPCR experiment using the GeNorm command in qbase+ software version 3.1 (Biogazelle, Zwijnaarde, Belgium—www.qbaseplus.com). An average *M* value of 0.796 was identified for *Gapdh* and *Hprt*, with *M* values up to 1 considered as acceptable stability for animal experiments.

### Statistical analysis

Body weight of animals and gene expression for the microarray validation were compared between the diet and LPS administration groups using a two-way ANOVA. Significant interactions were further analyzed by one-way ANOVA with a Tukey-Kramer post hoc test. Student’s *t* test was used to compare data between diet groups at each time point after LPS administration. Microarray data was analyzed in GeneSpring v13.1.1. Normalized expressions were analyzed via two-way ANOVA to test the effect of both LPS administration and diet and one-way ANOVA with a Tukey-Kramer post hoc test to examine the effect of LPS administration in each diet group. An unsupervised clustering was performed on genes that varied in the one-way ANOVA using a Pearson-centered correlation as a distance metric to build a hierarchical clustering heat map. The Venny online tool was used to identify overlap and unique genes between each post hoc list [34]. The results of each post hoc test was divided into positive or negative fold change (< 1.5), and a Benjamini and Yekutieli-corrected hypergeometric test (*p* < 0.1) was used to examine Gene Ontology (GO) functional category enrichment. GO categories were considered significant if they met the false discovery rate cutoff and contained at least two probe sets per category. Similar GO results were obtained when the samples were analyzed in the Database for Annotation, Visualization and Integrated Discovery (DAVID) version 6.8, an online bioinformatics tool offered by the National Institutes of Health (https://david.ncifcrf.gov/) [35]. A one-way ANOVA was performed on normalized expression values of genes driving categorical enrichment to examine main and interactive effects of LPS administration in each diet group. A *p* value < 0.05 (raw or false discovery rate corrected depending on the analysis) was considered statistically significant. The sample size was *n* = 7–8 for the body weight, *n* = 3 for the microarray data, and *n* = 5–8 for the microarray validation.

## Results

### Body weight

A significant main effect of LPS administration on body weight was detected at post-surgery days 1, 3, and 7 (*p* < 0.05, Additional file 1; Figure S2). The n-6 PUFA deprived and adequate groups were not significantly different from one another, with both groups losing approximately an average of 3 g. Mice fed with the n-6 PUFA deprived diet had lower body weight versus the adequate group at post-surgery day 14, regardless of LPS administration (*p* < 0.05, Additional file 1; Figure S2).

### Gene expression analysis

#### Microarray

**Fig. 1:**
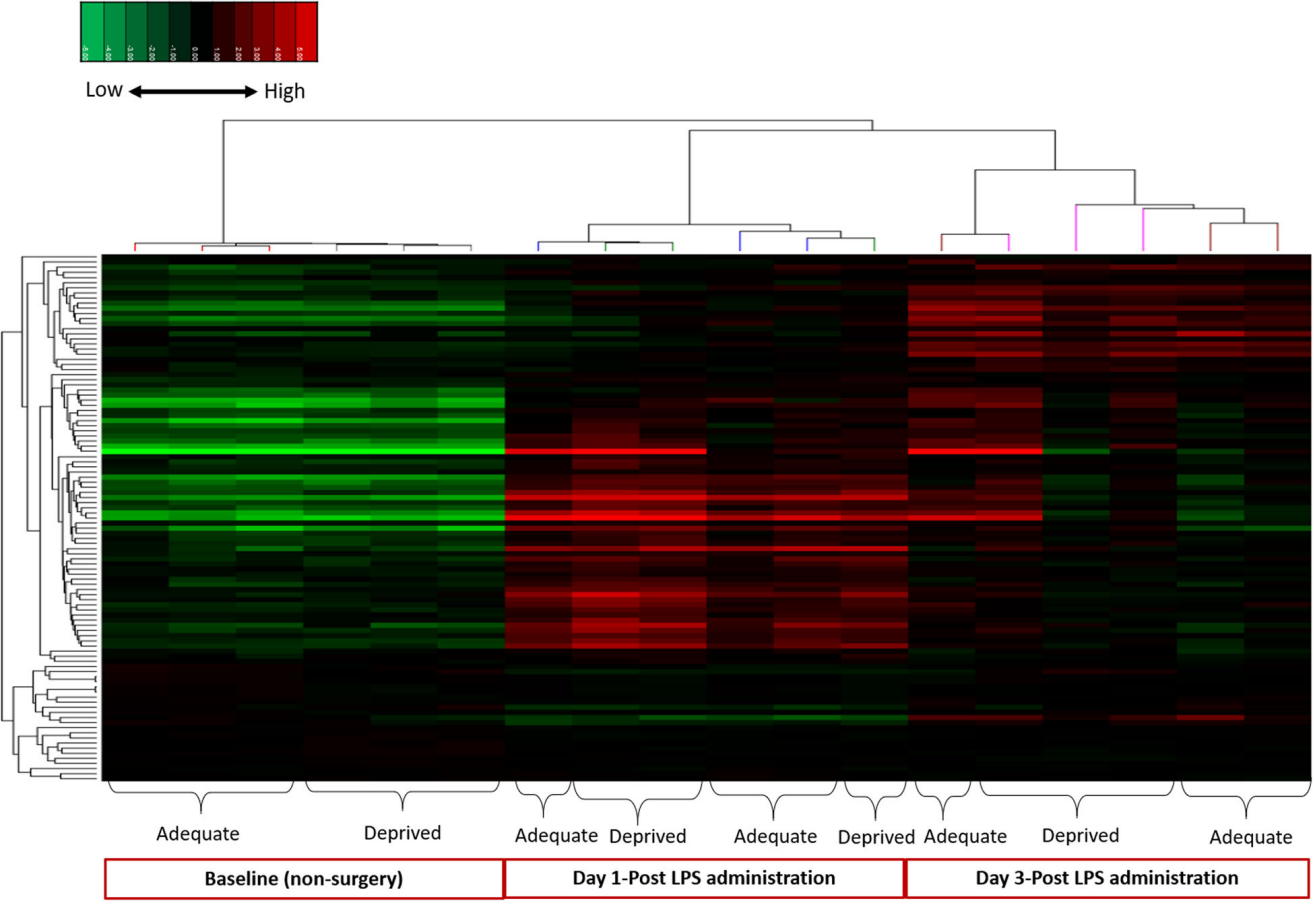
Hierarchical cluster of genes significantly increased in the one-way ANOVA (uncorrected *p* < 0.05, corrected *p* < 0.1). This clustering is zoomed in on key regions of clustering with labeled branches corresponding to individual samples; a clear separation is seen between baseline (non-surgery) and days after LPS administration. We scaled the expression intensities on rows (probe sets/genes) to make them weigh equally in the clustering. The colors of the heatmap are mapped linearly low expression in green and high expression in red. Adequate: n-6 PUFA adequate diet, deprived: n-6 PUFA deprived diet. *n* = 3 mice per group

The full microarray data (median centered) is presented in Additional file 2 (two-way ANOVA) and Additional file 3 (one-way ANOVA). Hierarchical clustering of genes found to be altered in a one-way ANOVA of the microarray data shows that samples cluster together by their respective surgery groupings (day 1 or day 3 post-surgery) regardless of their diet, indicating strong within surgery group similarities in gene expression patterns (Fig. 1). Although the low n-6 PUFA diet did not show an overall significant effect, there was a small effect between diet groups at baseline (corrected *p* value> 0.1). Irrespective of diet, 1 day following LPS administration exhibited more clustering of increased gene expression (red) for genes related to inflammation and immune processes than day 3 post-surgery, including Fc receptor (*Fcgr1*) and various components of lymphocytic molecules (lymphocytic antigen 86 (*Ly86*) and *Ly6a*), cluster of signaling protein differentiation and enzymes (signal transducer and activator of transcription 1 (*Stat1*), receptor transporter protein 4 (*Rtp4*), 2′-5′ oligoadenylate synthetase-like 1 (*Oasl2*), ubiquitin-specific peptidase 18 (*Usp18*), genes related to cytokine and chemokine signaling (*Irf7*, *Irf9*), interferon-induced transmembrane protein 3 (*Ifitm3*), interferon-induced protein with tetratricopeptide repeats 1 *(Ifit1*), and *Ccl12* (see Additional file 2 for the annotated cluster diagram). Similar trends of high expression of some genes such as the astrocyte marker (*Gfap*) complement component 1 q sub component, beta polypeptide (*C1qb*), beta-2 microglobulin (*B2m*), and a gene associated with n-6 PUFA metabolism cPLA_2_, group IVA (*Pla2g4a*), are presented at both 1 day and 3 days after LPS administration (see Additional file 2 for the annotated cluster diagram). Other genes such as colony stimulating factor 1 receptor (*Csfr1*), a component of immunoglobulin molecules (immunoglobulin heavy constant mu (*Ighm*)), and triggering receptor expressed on myeloid cells 2 (*Trem2*) are exclusively highly expressed at post-surgery day 3. The same genes appeared to be unchanged or downregulated in the non-surgery groups for the n-6 PUFA deprived and adequate fed animals.

**Fig. 2:**
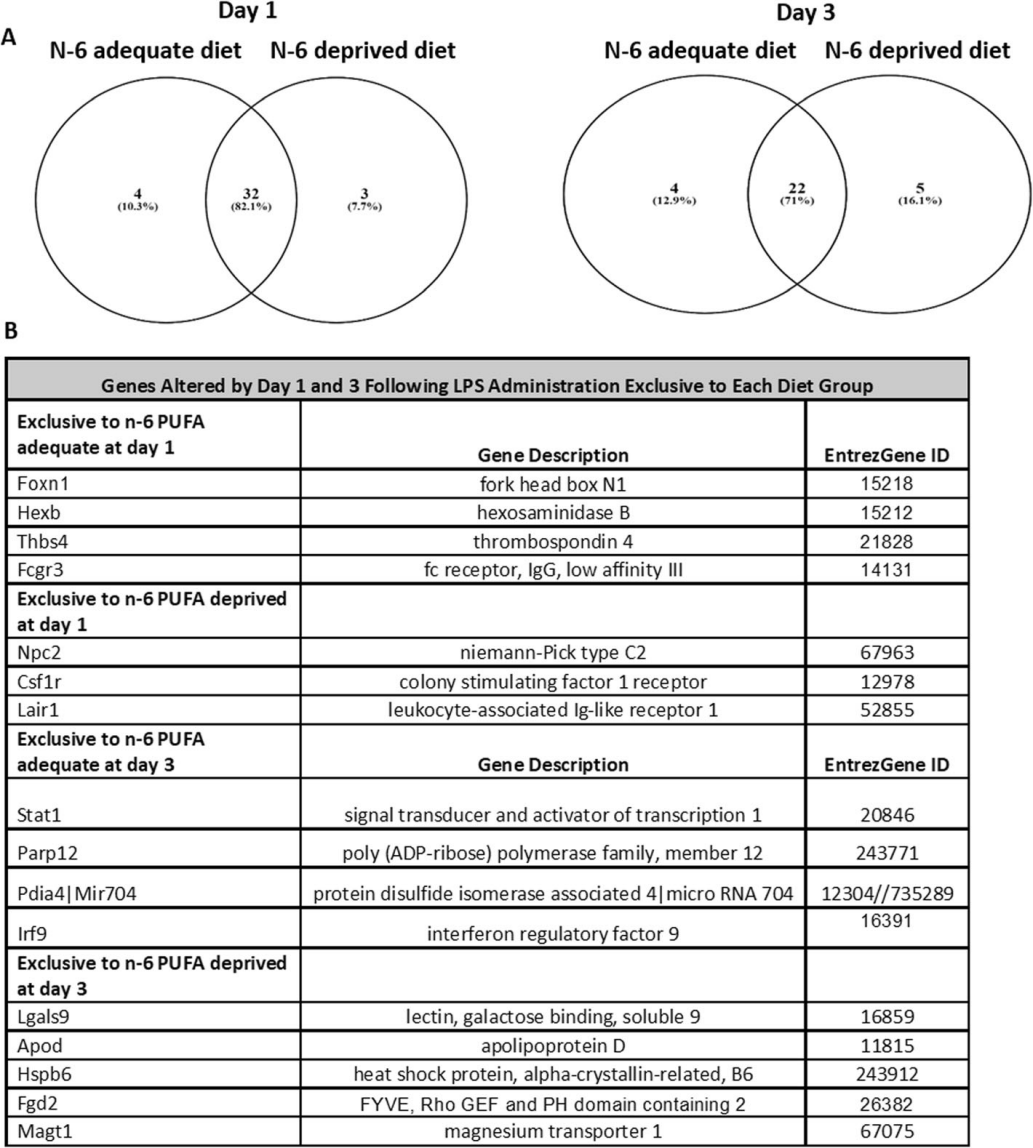
Analysis of the microarray data. **a** Venn diagram of genes increased by day 1 and day 3 post-LPS administration in each diet group. **b** List of genes increased by icv LPS administration in each diet group. *n* = 3 mice per group

LPS administered (1 day or 3 days post-surgery) and non-surgery animals within the n-6 PUFA deprived and adequate groups were compared post hoc following a one-way ANOVA to identify genes modified in response to the surgery in each diet group. After exclusion of un-indexed predicted genes, there were 32 overlapping probe sets altered by LPS administration between n-6 PUFA deprived and adequate groups at day 1 and 22 overlapping probes at day 3 following LPS administration. There were very few differently expressed genes between non-surgery and LPS-administered animals, with only four genes in the n-6 PUFA adequate, three in the n-6 PUFA deprived group at day 1, four genes in the n-6 PUFA adequate, and five in the n-6 PUFA deprived fed animals at day 3 (Fig. 2a, b, see Additional file 1 Tables S1 A and B for full gene lists). The majority of the shared genes, such as *Gfap*, *Ccl12*, *Irf7*, *Ly6a*, and *Fcgr1*, appeared to be more functionally important for response to LPS either at day 1 or day 3 in comparison to the ones that are exclusive to each diet. While *Aif1* (*iba1*), *Cd86*, and *Cd68*, as markers of the microglia, and *Ccl5*, a chemokine marker, were not significantly changed in the LPS-administered animals according to corrected *p* value of the microarray data (see Additional file 1, Figure S3), *Cd86*, *Cd68*, and *Ccl5* are highly expressed in response to LPS administration when considering the raw *p* value. Moreover, other inflammatory markers such as *Il-1β*, *Il-6*, *Tnfa*, and cyclooxygenase 2 (*Cox-2*; *Ptgs2*) did not show significant changes in response to LPS (see Additional file 1, Figure S3), when using either a corrected or raw *p* value. GO analysis was applied to the list of genes altered by LPS administration in each diet group to look for functional categories of gene expression altered in response to the day 1 and day 3 post-surgery. According to the post hoc comparison, the differences in genes between the n-6 PUFA adequate and deprived groups did not reach statistical significance and did not meet the fold change cutoff (more than 1.5) for GO analysis. Therefore, there was no categorical enrichment in response to diet.

The majority of the genes altered in response to LPS administration (day 1 and day 3) clustered significantly into functional categories after the false discovery rate correction in each diet group (Table 1 A and B for day 1 post-LPS administration, see Additional file 1 Tables S2 A and B for day 3 post-LPS administration). However, none of the exclusive genes, except for the *Stat1* gene that represents immune response enrichment, in the n-6 PUFA adequate or deprived group clustered significantly into functional categories after either raw or corrected *p* value. At day 1 post-surgery, the majority of the significant functional enrichments (~ 75%) were related to immune system activation, such as antigen processing and presentation of peptide antigen, immunoglobulin-mediated immune response, or regulation of immune system process, while only about 30% of the enrichment was seen at day 3 of post-LPS administration was involved in immune- and inflammatory-related processes. Most of the remaining categories at each time point after LPS administration are parent categories upstream of immune-related categories, such as response to external stimulus or defense response.

**Table 1:** List of significantly enriched Gene Ontology categories in (A) n-6 PUFA adequate and (B) n-6 PUFA deprived at day 1 LPS-administered mice compared to non-surgery mice

GO term	BY-corrected *p* value	No. of genes driving enrichment	GO term	BY-corrected *p* value	No. of genes driving enrichment
A. Significantly enriched GO categories in n-6 PUFA adequate at baseline (non-surgery) vs day 1 LPS-administered mice					
Immune response	1.18E−06	11	Regulation of immune response	0.05	5
Innate immune response	1.18E−06	9	Regulation of adaptive immune response based on somatic recombination of immune receptors built from immunoglobulin superfamily domains	0.05	3
Immune system process	2.12E−05	12	Immunoglobulin-mediated immune response	0.05	3
Cellular response to type I interferon	1.90E−04	3	Negative regulation of neuron projection development	0.05	3
Type I interferon signaling pathway	1.90E−04	3	B cell-mediated immunity	0.05	3
Defense response	3.52E−04	9	Regulation of adaptive immune response	0.06	3
Response to type I interferon	3.75E−04	3	Regulation of response to stimulus	0.06	10
Antigen processing and presentation of peptide antigen	7.9E−04	4	Defense response to virus	0.06	3
Immune effector process	1.84E−03	6	Extracellular space	0.06	7
Response to stress	1.84E−03	12	Viral process	0.06	4
Glycoprotein binding	2.67E−03	4	Multi-organism cellular process	0.06	4
Neuron projection regeneration	2.67E−03	3	Negative regulation of cell projection organization	0.07	3
Response to interferon-beta	4.35E−03	3	Regulation of immune effector process	0.07	4
Antigen processing and presentation	5.28E−03	4	Regulation of leukocyte mediated immunity	0.08	3
Response to external biotic stimulus	5.46E−03	7	Extracellular matrix organization	0.08	3
Response to other organism	5.46E−03	7	Extracellular structure organization	0.08	3
Response to biotic stimulus	6.68E−03	7	Regeneration	0.08	3
Antigen processing and presentation of peptide antigen via MHC class I	0.01	3	Negative regulation of cytokine production	0.08	3
Regulation of multi-organism process	0.01	5	Lymphocyte-mediated immunity	0.08	3
Response to stimulus	0.01	17	Adaptive immune response based on somatic recombination of immune receptors built from immunoglobulin superfamily domains	0.08	3
Regulation of response to external stimulus	0.01	6	Positive regulation of immune system process	0.08	5
Negative regulation of multicellular organismal process	0.01	7	Regulation of response to biotic stimulus	0.08	3
Response to virus	0.01	4	Humoral immune response	0.08	3
Positive regulation of immune response	0.01	5	Symbiosis, encompassing mutualism through parasitism	0.08	4
Defense response to other organism	0.02	5	Interspecies interaction between organisms	0.08	4
Regulation of immune system process	0.02	7	Positive regulation of response to stimulus	0.08	7
Positive regulation of response to external stimulus	0.02	4	Protein complex binding	0.08	5
Regulation of inflammatory response	0.02	4	Regulation of localization	0.09	8
Regulation of defense response	0.03	5	Leukocyte-mediated immunity	0.09	3
Regulation of transport	0.03	8	Response to external stimulus	0.09	7
Negative regulation of cellular component organization	0.04	5	Negative regulation of neuron differentiation	0.09	3
Adaptive immune response	0.04	4	Negative regulation of protein transport	0.09	3
Regulation of lymphocyte mediated immunity	0.05	3			
B. Significantly enriched GO categories in deprived n-6 PUFA at baseline (non-surgery) vs day 1 LPS-administered mice					
Innate immune response	5.95E−08	11	Regulation of defense response	0.02	6
Immune response	9.19E−08	13	Antigen processing and presentation of peptide antigen via MHC class I	0.02	3
Immune system process	6.45E−07	15	Lymphocyte-mediated immunity	0.02	4
Defense response	5.82E−05	11	Adaptive immune response based on somatic recombination of immune receptors built from immunoglobulin superfamily domains	0.02	4
Type I interferon signaling pathway	4.14E−04	3	Response to stimulus	0.02	20
Cellular response to type I interferon	4.14E−04	3	Regulation of immune response	0.03	6
Immune effector process	9.36E−04	7	Regulation of multi-organism process	0.03	5
Response to type I interferon	9.36E−04	3	Response to virus	0.03	4
Response to stress	2.01E−03	14	Response to interferon gamma	0.03	3
Antigen processing and presentation of peptide antigen	2.01E−03	4	Leukocyte-mediated immunity	0.03	4
Regulation of immune system process	6.05E−03	9	Positive regulation of defense response	0.04	4
Neuron projection regeneration	6.05E−03	3	Positive regulation of response to stimulus	0.04	9
Response to external biotic stimulus	6.05E−03	8	Regulation of transport	0.04	9
Response to other organism	6.05E−03	8	Defense response to other organism	0.04	5
Response to biotic stimulus	6.78E−03	8	Regulation of response to stimulus	0.04	12
Glycoprotein binding	7.16E−03	4	Positive regulation of response to external stimulus	0.05	4
Positive regulation of immune response	7.40E−03	6	Regulation of inflammatory response	0.05	4
Regulation of response to external stimulus	7.65E−03	7	Negative regulation of multicellular organismal process	0.05	7
Response to interferon-beta	7.65E−03	3	G protein-coupled receptor binding	0.06	4
Regulation of lymphocyte migration	7.65E−03	3	Regulation of cytokine production	0.06	5
Immunoglobulin-mediated immune response	8.89E−03	4	Extracellular space	0.07	8
B cell-mediated immunity	9.05E−03	4	Regulation of lymphocyte-mediated immunity	0.08	3
Antigen processing and presentation	0.01	4	Cytokine-mediated signaling pathway	0.08	4
Regulation of adaptive immune response	0.01	4	Regulation of adaptive immune response based on somatic recombination of immune receptors built from immunoglobulin superfamily domains	0.08	3
Positive regulation of immune system process	0.01	7	Negative regulation of cellular component organization	0.08	5
Adaptive immune response	0.01	5	Negative regulation of neuron projection development	0.09	3
			Regulation of multicellular organismal process	0.10	10

Based on *n* = 3 mice per group

*BY* Benjamini Yekutieli false discovery rate, *GO* Gene Ontology

**Fig. 3:**
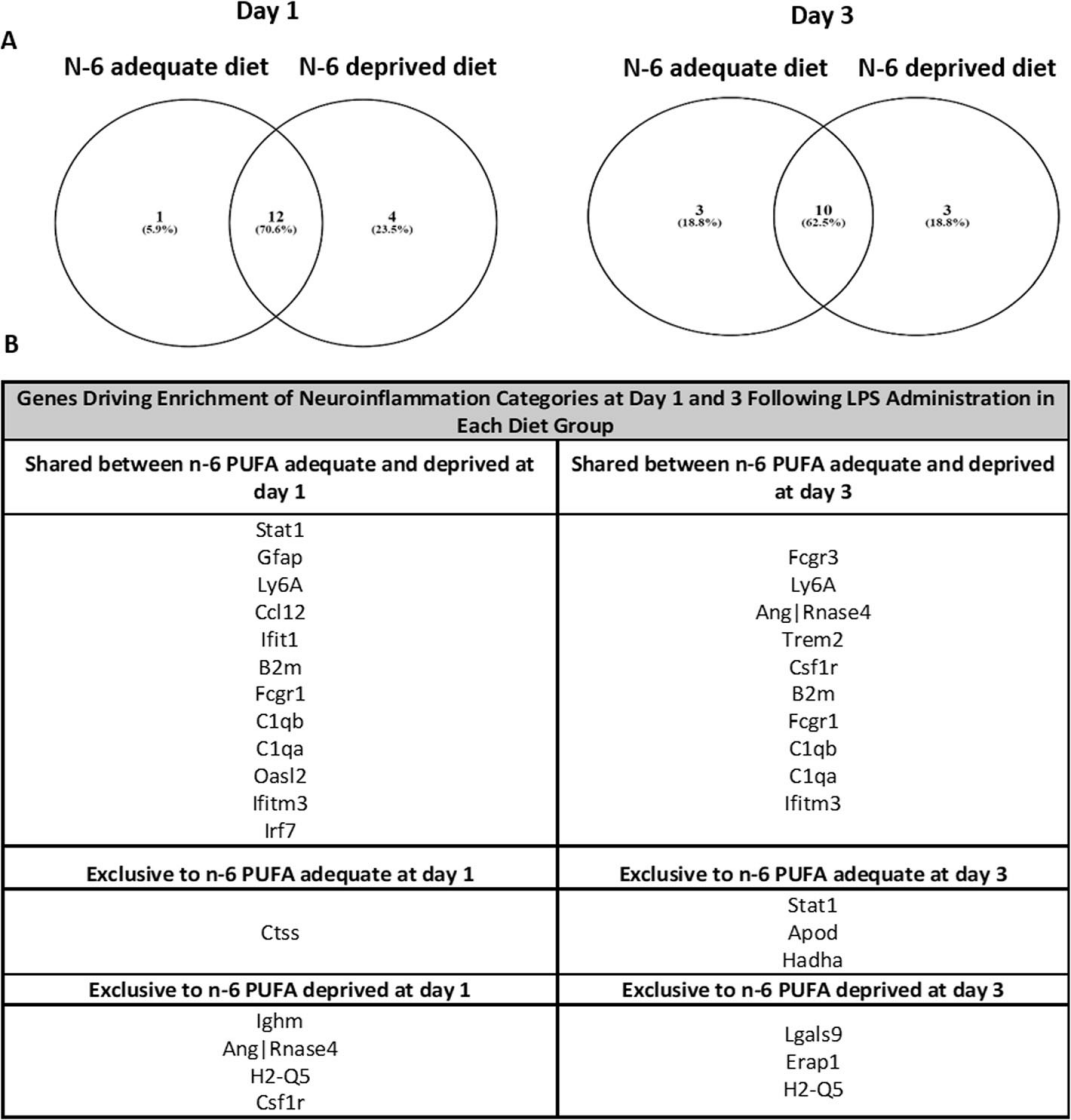
Analysis of Gene Ontology. **a** Venn diagram of genes driving enrichment of inflammation categories. **b** List of genes driving enrichment of inflammation-associated gene expression categories at day 1 and day 3 post-LPS administration in each diet group. *n* = 3 mice per group

**Fig. 4:**
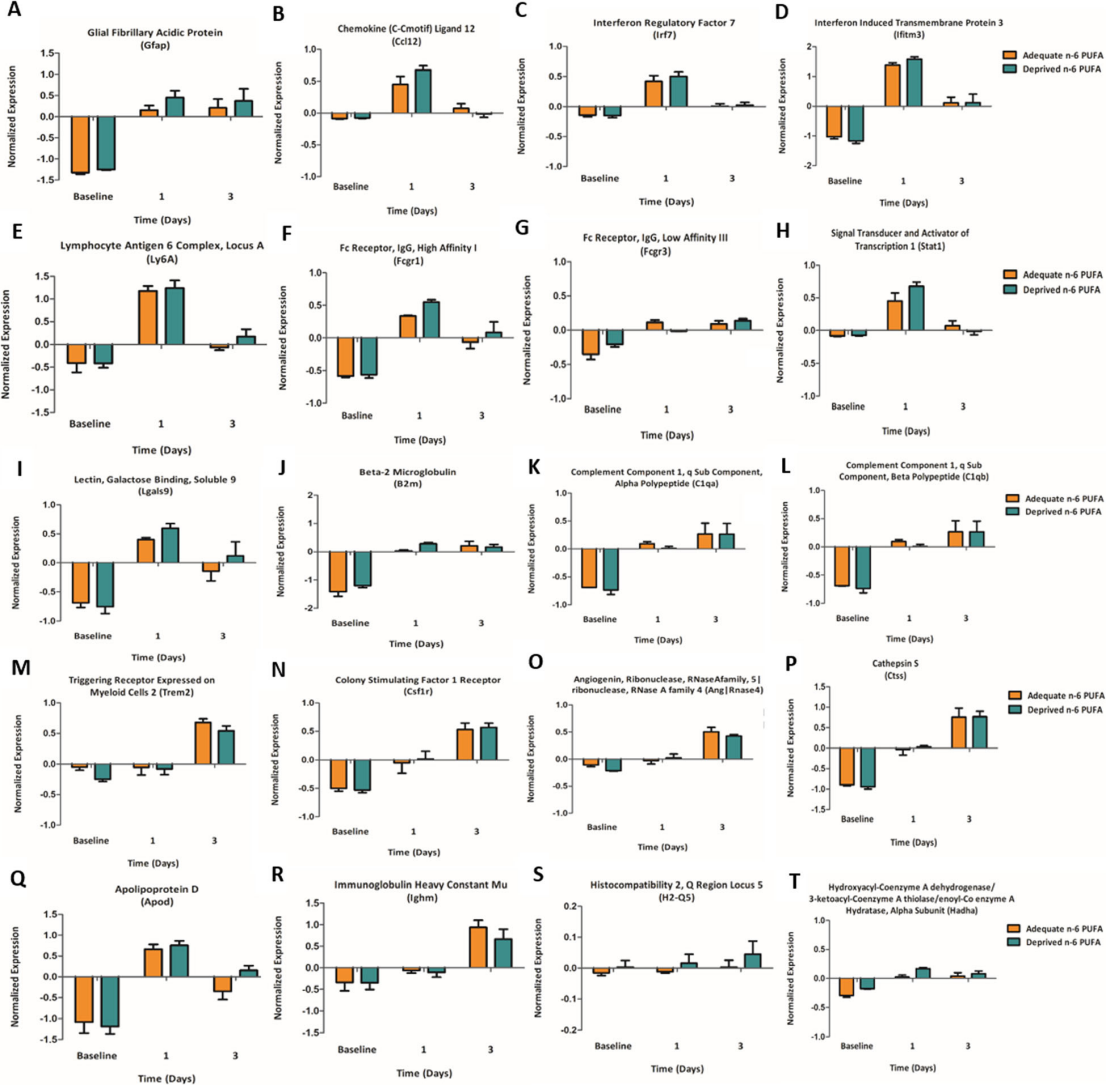
Genes driving enrichment of inflammation-associated gene expression categories at day 1 and day 3 post-LPS administration in the n-6 PUFA deprived and adequate fed mice. **a** *Gfap*, glial fibrillary acidic protein. **b** *Ccl 12*, chemokine(C-Cmotif) ligand 12. **c** *Irf7*, interferon regulatory factor 7. **d** *Ifitm3*, interferon-induced transmembrane protein 3. **e** *Ly6a*, lymphocytic antigen 6 complex, locus A. **f** Fcgr1, Fc receptor IgG high affinity 1 gamma polypeptide. **g** *Fcgr3*, Fc receptor IgG, low affinity III. **h** *Stat1*, signal transducer and activator of transcription 1. **i** *Lgals9*, lectin, galactose binding, soluble 9. **j** *B2m*, beta-2 microglobulin. **k** *C1qa*, complement component 1 q sub component, alpha polypeptide. **l** *C1qb*, complement component 1 q sub component, beta polypeptide. **m** *Trem2*, triggering receptor expressed on myeloid cells 2. **n** *Csf1r*, colony stimulating factor 1 receptor. **o** *Ang|Rnase4*, angiogenin, 5|ribonuclease, RNase A family 4. **p** *Ctss*, cathepsin S. **q** *Apod*, apolipoprotein D. **r** *Ighm*, immunoglobulin heavy constant mu. **s** MHC l: *H2-Q5*, histocompatibility 2, Q region locus 5. **t** *Hadha*, hydroxyacyl-Coenzyme A dehydrogenase/3-ketoacyl-Coenzyme A thiolase/enoyl-Coenzyme A hydratase (tri functional protein), alpha subunit. Gene names are provided with common name and abbreviated gene name in brackets. Bars represent means ± standard error of the mean, *n* = 3 mice per group. Major histocompatibility complex (MHC). Baseline refers to non-surgery; 1 and 3 refers to day 1 and day 3 post-LPS administration

Increased expression of 16 and 13 genes drove this categorical enrichment at day 1 of LPS administration in the n-6 PUFA deprived and adequate groups, respectively, with 12 genes overlapping, while 13 genes (10 overlapped) were involved at day 3 post-surgery for each diet (Fig. 3a, b). Many of those genes are highlighted in the hierarchical cluster analysis: *Gfap* (Fig. 4a), *Ccl12* (Fig. 4b), *Irf7* (Fig. 4c), *Ifitm3* (Fig. 4d), *Ly6a* (Fig. 4e), *Fcgr1* (Fig. 4f), Fc receptor, IgG, low affinity III (Fig. 4g, *Fcgr3*), *Stat1* (Fig. 4h), lectin, galactose binding, soluble 9 (Fig. 4i, Lgals9), *B2m* (Fig. 4j), complement component 1 q sub component, alpha polypeptide (Fig. 4k, *C1qa*), *C1qb* (Fig. 4l), *Trem2* (Fig. 4m), *Csf1r* (Fig. 4n), angiogenin, 5|ribonuclease, RNase A family 4 (Fig. 4o, *Ang|Rnase4*), cathepsin S (Fig. 4p, *Ctss*), apolipoprotein D (Fig. 4q, *Apod*), *Ighm* (Fig. 4r), MHC ll: major histocompatibility 2, Q region locus 5 (Fig. 4s, *H2-Q5*), hydroxyacyl-Coenzyme A dehydrogenase/3-ketoacyl-Coenzyme A thiolase/enoyl-Coenzyme A hydratase (tri functional protein), and alpha subunit (Fig. 4t, *Hadha*). cPLA_2_, a gene involved in n-6 PUFA metabolism, is also one of the genes that were identified in the hierarchical map; however, it did not drive any categorical enrichment between non-surgery and LPS administration groups. All *p* values for individual genes remained significant after the false discovery rate correction. Post hoc tests for all genes revealed higher expression in the LPS-administered mice than non-surgery animals at both time points, regardless of diet group (Fig. 4).

**Fig. 5:**
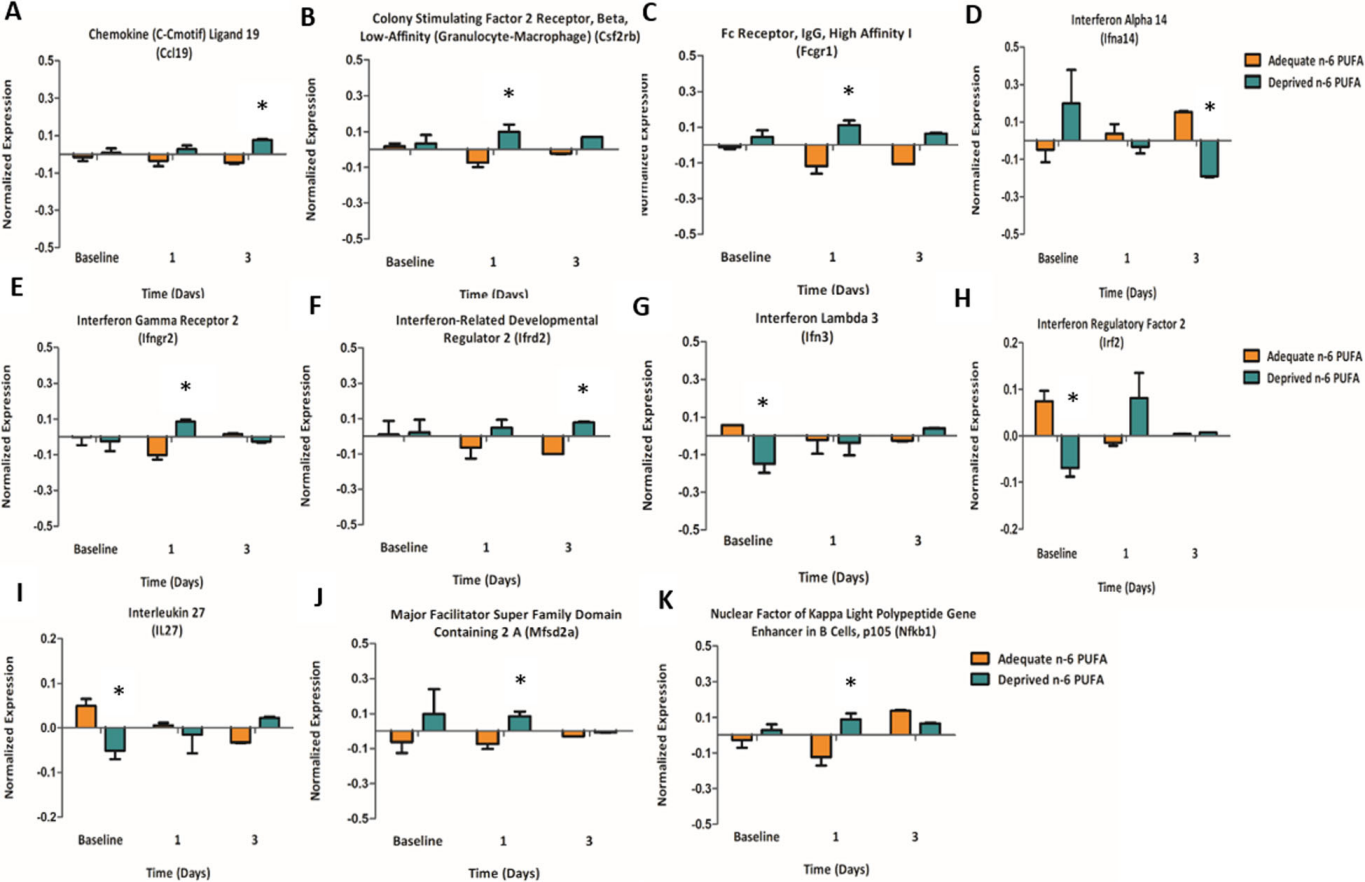
Genes involved in inflammation in response to n-6 PUFA deprived versus adequate diet using *t* test data (*p* < 0.01) of the microarray. **a** *Cc19*, chemokine (C-Cmotif) ligand 19. **b** *Csf2rb*, colony stimulating factor 2 receptor, beta, low affinity (granulocyte-macrophage). **c** *Fcgr1*, Fc receptor IgG high affinity 1 gamma polypeptide. **d** *Ifna14*, interferon alpha 14. **e** *Ifngr2*, interferon gamma receptor 2. **f** *Ifrd2*, interferon-related developmental regulator 2. **g** *Ifnl3*, interferon lambda 3. **h** *Irf2*, interferon regulatory factor 2. **i** *IL27*, interleukin 27. **j** *Mfsd2a*, major facilitator super family domain containing 2 A. **k** *Nfkb1*, nuclear factor of kappa light polypeptide gene enhancer in B cells, p105. Asterisks indicate significant effect of n-6 PUFA deprived diet compared to n-6 PUFA adequate diet

Although gene expression was not significantly different between any of the diet groups following two- and one-way ANOVA (considering either raw or corrected *p* value), it was of interest to conduct an exploratory analysis of the candidate genes in response to the n-6 PUFA deprived versus adequate diet using Student’s *t* test of microarray data at baseline, day 1, and day 3 (see Additional file 4). The genes with a cutoff raw (uncorrected) *p* value of < 0.01, such as *Ccl19*, *Ifrd2*, *Ifngr2*, *Irf2*, and *Mfsd2a*, were identified and validated by qPCR. Comparison of the normalized expression of these genes between diet groups with an uncorrected *t* test identified significant diet effects for many genes involved in inflammatory and immune processes at baseline (non-surgery), day 1, or day 3 post-surgery (Fig. 5). The expression of other common inflammation-associated genes of microglia, cytokines, and chemokine markers, such as *Aif1*, *Cd86*, *Cxcl11*, and *Il-6ra* and genes involved in the synthesis of n-6 PUFA pro-inflammatory mediators (arachidonate 15 lipoxygenase, *Alox15*), were changed in response to an n-6 PUFA deprived diet; however, they did not meet the raw (uncorrected) *p* value cutoff of < 0.01 and thus were not used for validation (Additional file 4).

#### Microarray validation

**Fig. 6:**
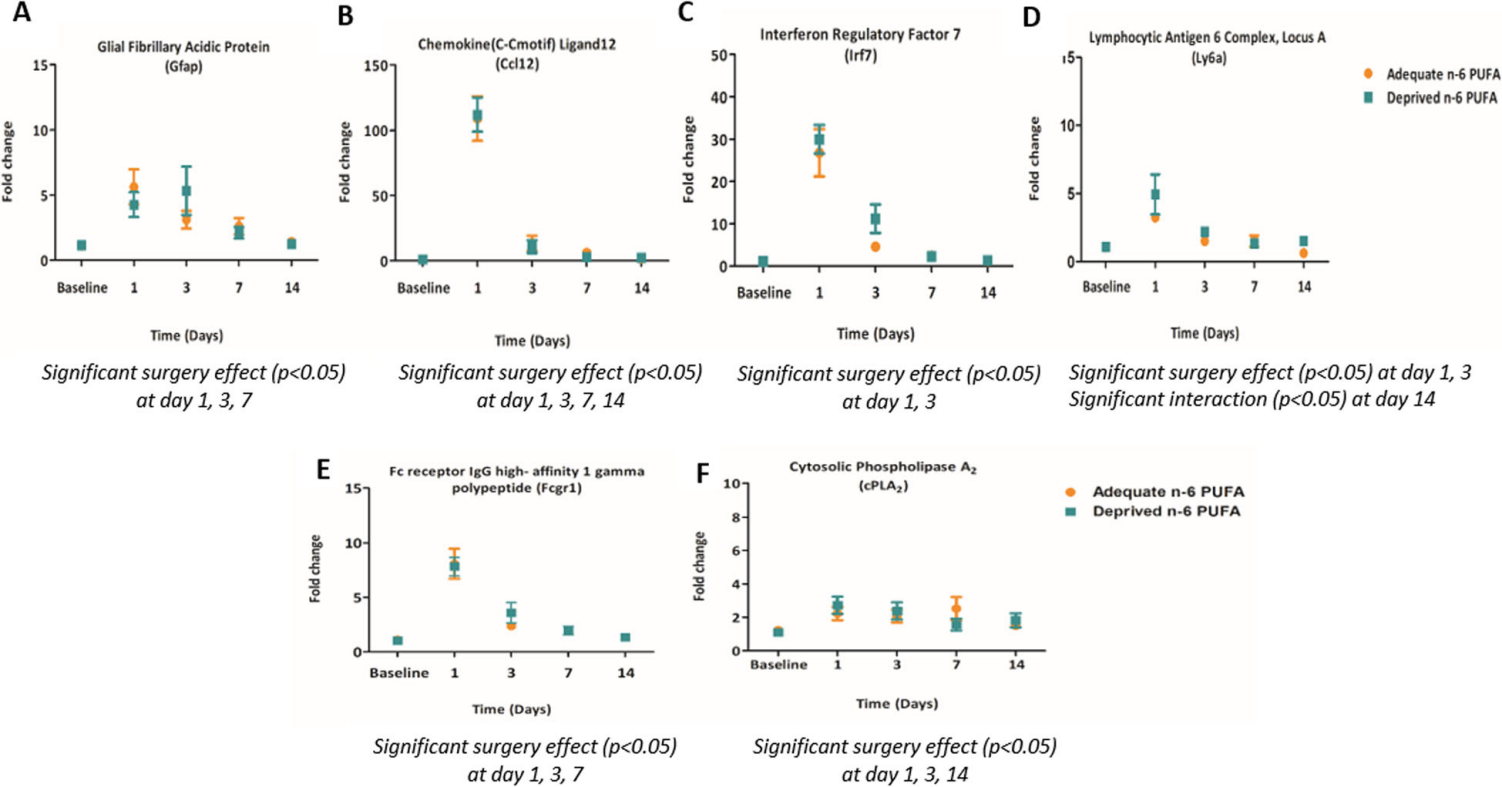
Validation of a subset of genes driving the enrichment of inflammation-associated gene expression categories as well as n-6 PUFA metabolism in the microarray. **a** *Gfap*, glial fibrillary acidic protein. **b** *Ccl 12*, chemokine(C-Cmotif) ligand 12. **c** *Irf7*, interferon regulatory factor 7. **d** *Ly6a*, lymphocytic antigen 6 complex, locus A. **e** *Fcgr1*, Fc receptor IgG high affinity 1 gamma polypeptide. **f** cPLA_2_ group IVA (*Pla2g4a*), cytosolic phospholipase A_2_. Graphs represent mean ± standard error of the mean, *n* = 5–8 mice per group

To validate the Gene Ontology results for LPS surgery, expression of *Fcgr1*, *Gfap*, *Irf7*, *Ly6a*, *Ccl12*, and *Pla2g4a* was measured by RT-qPCR at days 1, 3, 7, and 14 post-LPS administration, as we were interested in the time course of genes expressions and in an independent cohort of non-surgery animals. When normalized to baseline (non-surgery) via the relative expression 2^−ΔΔCt^, LPS-administered animals exhibited increased expression of all the previous genes compared to non-surgery mice at days 1 and 3 (Fig. 6), confirming and extending the microarray results. Some gene expressions, including *Irf7* (Fig. 6c), *Ly6a* (Fig. 6d), and cPLA_2_ (*Pla2g4a*) (Fig. 6f), appear to be decreased at 7 days while *Fcgr1* (Fig. 6e) and *Gfap* (Fig. 6a) decreased at 14 days following LPS administration in comparison to non-surgery animals. However, *Ccl12* (Fig. 6b) expression remained to be elevated at all time points post-LPS administration. While not being affected by LPS surgery at day 14, *Ly6a* signal appears to be upregulated in the n-6 PUFA deprived versus adequate fed animals (*p* = 0.005). Interestingly, *Irf7* and *Ly6a*, cytokine and immune cell markers, respectively, were also elevated in the n-6 PUFA deprived in comparison to n-6 PUFA adequate at day 3 and day 14, respectively, using a *t* test.

**Fig. 7:**
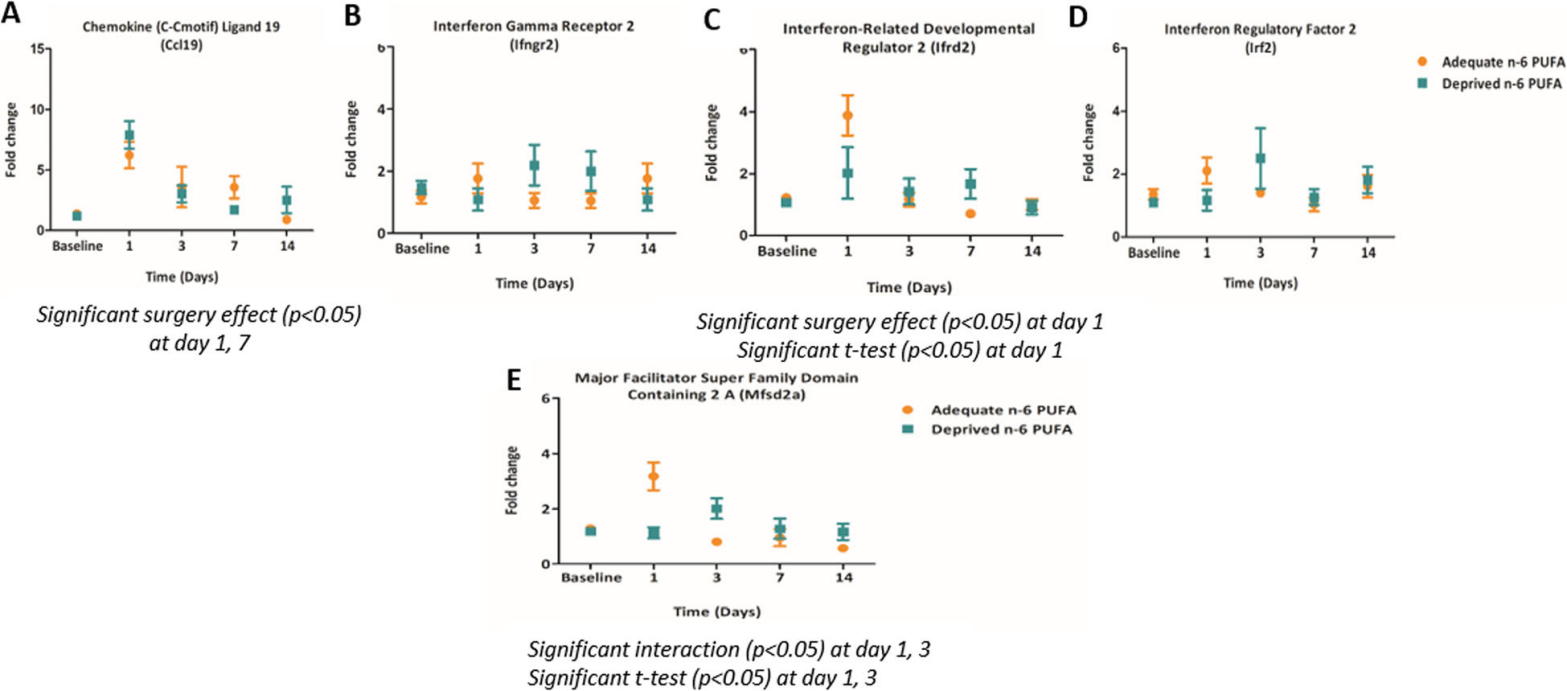
Validation of a subset of genes involved in inflammation in response to n-6 PUFA deprived versus adequate diet in the microarray. **a** *Cc19*, chemokine (C-Cmotif) ligand 19. **b** *Ifngr2*, interferon gamma receptor 2. **c** *Ifrd2*, interferon-related developmental regulator 2. **d** *Irf2*, interferon regulatory factor 2. **e** *Mfsd2a*, major facilitator super family domain containing 2 A. Graphs represent mean ± standard error of the mean, *n* = 5–8 mice per group

To validate the uncorrected *t* test results in response to diet, expression of the five genes, *Ccl19*, *Irf2*, *Ifrd2*, *Ifngr2*, and *Mfsd2a*, were measured at the same time points following LPS administration in both diet treatments (Fig. 7). Comparison of the gene expression between the LPS administration and diet groups via two-way ANOVA identified that *Ccl19* and *Ifrd2*, chemokine and cytokine markers, expressions were increased at day 1, and day 7 for *Ccl19* only, following LPS administration. Similarly, *Mfsd2a* was also elevated at day 1 and day 3 after LPS administration. Moreover, *Mfsd2a* was decreased at day 1 and increased at day 3 in the n-6 PUFA deprived versus adequate group.

Microarray validation did not confirm inflammatory expression in response to the n-6 PUFA deprived diet (Fig. 7). Contrary to the microarray uncorrected *t* test results, the genes were not significantly higher in the animals fed n-6 PUFA deprived diet versus n-6 PUFA adequate diet either at baseline, day 1, and/or day 3, except for *Ifrd2* (Fig. 7c, *p* < 0.05) and Mfsd2a (Fig. 7e, *p* < 0.05). *Ifrd2* and *Mfsd2a* had lower expression in response to n-6 PUFA deprived diet at day 1 after surgery. However, *Mfsd2a* was significantly increased in the n-6 PUFA deprived at day 3 post-LPS surgery. There was also a decrease in the expression of *Irf2* (Fig. 7d) at day 1 (*p* = 0.06) and *Ccl19* (Fig. 7a) at day 7 and an increase in *Ifrd2* (Fig. 7c) at day 7 in the n-6 PUFA deprived fed animals after LPS administration, but they did not reach statistical significance (*p* = 0.07). Moreover, none of the groups differed significantly in expression of Ifngr2 (Fig. 7b), though a similar pattern of *Ifrd2* and *Mfsd2a* expression was observed.

## Discussion

This study is the first to perform a microarray in response to neuroinflammation and its resolution in relation to lowering dietary n-6 PUFA. Although mice consuming the n-6 PUFA deprived diet had lower body weight versus the adequate group at post-surgery day 14, regardless of LPS administration, the magnitude of this difference was small and consistent with another report [12]. We saw an upregulation of pro-inflammatory and lipid-related genes after LPS administration, in agreement with previous studies [6–8, 26]. Increasing pro-inflammatory gene expressions were associated with gene inflammation categories in response to icv administration of LPS. However, these pro-inflammatory markers were not reduced by lowering dietary n-6 PUFA as previously hypothesized. A recent review shows that in studies reporting an effect on the brain n-6 PUFA, LA accounted for on average 30% difference of dietary fatty acids between n-6 PUFA deprived and adequate diets [36]; the difference in LA levels between both diets in a study similar to what we report here was ~ 20% [13], which may not be big enough to show major changes in inflammatory markers. Another possible reason for the minor changes in pro-inflammatory markers over time between diet groups is that the diet exposure (∼ 9 weeks) is shorter compared to those of previous studies [26], which were about 15 weeks of intervention.

It has been proposed that DHA might exert protective effects in neuroinflammation, in part, via competition with ARA metabolism. Our current study, where we have previously reported brain ARA and lipid mediators are substantially decreased [13], does not support that lower ARA is robustly protective in neuroinflammation, albeit we cannot rule out an interaction between increased DHA and decreased ARA. In a previous work from our group, administering DHA or ARA icv suggested that DHA can exert anti-neuroinflammatory effects independent of ARA [8, 12, 24]. Interestingly, lowering n-6 PUFA from 7.4 to 2.4% of energy and increasing n-3 PUFA intake to 1.5 g/day for 12 weeks reduced headache frequency in chronic headache patients [37]. In these subjects, the frequency of headaches was positively associated with total linoleic acid concentrations and levels of pro-inflammatory mediators 8- and 9-hydroxyeicosatetraenoic acids produced from ARA as well as 11-hydroxy-12,13-trans-epoxy-(9Z)-octadecenoate released from linoleic acid, in plasma [38, 39]. Although other inflammatory markers (cytokines, chemokines, microglia) have not been examined, Ramsden et al. demonstrated that increasing dietary n-3 PUFA concurrent with decreasing n-6 PUFA has more pronounced effects in comparison to only lowering n-6 PUFA. In this present study, both diet treatments provide equal amounts of n-3 PUFA ~ 3.4% of total fatty acids (~ 0.4% of energy); thus, it might be another reason why lowering n-6 PUFA resulted in subtle changes in response to LPS administration. A previous study in rats reported that lowering n-6 PUFA reduced pro-inflammatory gene expression of Cox-2 and Pge2 after LPS administration in brain [26], which was not observed in our study. However, there were many methodological differences between these studies.

Six genes that were selected for qPCR validation in response to LPS administration are either expressed on astrocytes (*Gfap*), both astrocytes and microglia (*Fcgr1*), lymphocytes (*Ly6A*), and inflammatory cytokines and chemokines (*Irf7*, *Ccl12*) or are involved in n-6 PUFA metabolism (*Pla2g4a*). All these genes, except *Pla2g4a*, were selected according to their enrichment effect in driving the Gene Ontology analysis including immune system process, innate immune response, and phagocytosis. The other five genes were based on the most significant uncorrected *p* value from the microarray analysis in response to n-6 PUFA diet. *Ifrd2*, *Ifngr2*, *Irf2*, *and Ccl19* are inflammatory cytokines and chemokines while Mfsd2a is thought to be a blood-brain barrier (BBB) transport protein [40]. Studies show that there is a positive correlation between the numbers of astrocytes and *Mfsd2a* expression in the brain [41]. Thus, it is possible that this upregulation of *Mfsd2a* in n-6 PUFA adequate fed mice is due to the high presence of astrocytes that maintain expression of Mfsd2a in the brain and control BBB homeostasis. The gene expression of *Gfap* has a similar trend of *Mfsd2a* expression as shown in microarray validation. *Gfap* had maximum expression at day 1 after LPS administration in the n-6 PUFA adequate fed mice followed by high expression at day 3 post-surgery in the n-6 PUFA deprived group. Future studies in Mfsd2a knock-out mice are needed to fully understand the mechanism between n-6 PUFA and Mfsd2a gene expression in the brain. All the classical inflammatory markers related to microglia, cytokines, and chemokines in the microarray analysis were not significantly elevated according to the corrected *p* value; however, the majority of them were statistically significant in response to LPS administration using a raw (uncorrected) *p* value (see Additional file 1, Figure S3). However, the common genes associated with inflammation, such as *Aif1* (*iba1*), *Cd86*, *Cd68*, *Il-1β*, *IL6ra*, *Tnfaip6*, *Ptgs2* (*Cox-2*), and *Ccl5*, in response to LPS administration were not used for validation because they did not drive the Gene Ontology enrichment (non-significant according to the corrected *p* value); in addition, they were not driving the hierarchical clustering of samples, while in response to an n-6 PUFA diet, the expression of these inflammatory genes did not reach the raw *p* value cutoffs (< 0.01), and thus were not used for validation.

The microarray study was hypothesis generating, seeking genes and gene expression categories that could be differentially affected by the diet groups in response to icv. Furthermore, the microarray was underpowered and caution must be taken when interpreting any results as null. In contrast, the validation study was hypothesis testing and was better powered to identify differences between the groups (*n* = 5–8). A limitation of this study is that the sample size is relatively small for microarray analysis and may not be enough to detect subtle changes induced by n-6 PUFA diets, especially when using a corrected *p* value. Using non-surgery animals as a control group is also another limitation, although we have previously reported that LPS induces a more robust inflammatory response than icv injection of vehicle [8]. Moreover, additional markers would be needed to make more comprehensive conclusions about dietary n-6 PUFA modulation and neuroinflammation. Another limitation of this work is that some markers are induced within hours (~ 8 h) following LPS administration [7]; thus, it is possible that we may have missed changes in expression of some genes before day 1 of LPS administration.

## Conclusions

The n-6 PUFA deprived diet modulated (both increased and decreased) the expression of some of the inflammatory genes, but it did not delay the resolution response to LPS relative to an n-6 PUFA adequate diet over the time course of inflammation. Although lowering n-6 PUFA have been shown in some human observational and animal studies to reduce inflammatory markers in the periphery, these effects were relatively minor in the hippocampus. Further research testing dietary n-6 PUFA modulation on a variety of brain inflammatory responses is required to determine the role of dietary n-6 PUFA in regulation of neuroinflammation.

## Supplementary information

**Additional file 1: Figure S1.** Stability of various common reference (housekeeping) genes across diet/surgery groups. Glyceraldehyde 3-phosphate dehydrogenase (*Gapdh*) and hypoxanthine guanine phosphoribosyltransferase (*Hprt*). Figures represent *n* = 5–8 mice per group. **Figure S2.** Body weights of animals fed n-6 PUFA deprived versus n-6 PUFA adequate before LPS administration and at 1, 3, 7 and 14 days after LPS administration. Bars represent mean ± standard error of the mean of *n* = 7–8 mice per group. **Figure S3.** Genes involved in the inflammatory response to LPS administration using a one-way ANOVA (corrected *p* > 0.05) of the microarray. A) *Aif1*: allograft inflammatory factor 1, B) *Cd86*: cluster of differentiation 86 antigen, C) *Cd68*: cluster of differentiation 68 antigen, D) *IL-1β*: interleukin 1 beta, E) *IL-6ra*: interleukin 6 receptor alpha chain, F) *Tnf-aip6*: tumor necrosis factor alpha induced protein 6, G) *Ptgs2* (*Cox-2*): prostaglandin-endoperoxide synthase 2, and H) *Ccl5*: chemokine (C-C motif) ligand 5. Bars represent mean ± standard error of the mean of *n* = 3 mice per group. Significant differences between LPS (day 1 and day 3) and baseline (non-surgery) groups are represented by * (raw *p* < 0.05). **Table S1.** List of genes altered by (A) day 1 and (B) day 3 after LPS administration in each diet group. Figures represent *n* = 3 mice per group. **Table S2.** List of significantly enriched gene ontology categories in (A) n-6 PUFA adequate and (B) n-6 PUFA deprived at day 3 LPS-administered compared to non-surgery mice. Based on *n* = 3 mice per group. Benjamini Yekutieli false discovery rate (BY), Gene ontology (GO).

**Additional file 2.** The full microarray data (median centered) is presented in two-way ANOVA analysis. N-6 PUFA adequate and n-6 PUFA deprived diet groups at baseline, day 1, and day 3 following LPS administration.

**Additional file 3.** The full microarray data (median centered) is presented in one-way ANOVA analysis. N-6 PUFA adequate and n-6 PUFA deprived diet groups at baseline, day 1, and day 3 following LPS administration.

**Additional file 4.** An exploratory analysis of the candidate genes in response to the n-6 PUFA deprived versus adequate diet using Student’s t-test of microarray data at baseline, day 1, and day 3 following LPS administration.

## Data Availability

Data and, where available, materials can be provided for non-commercial purposes upon request to the corresponding author.

## Abbreviations

AD: Alzheimer’s disease
ANOVA: Analysis of variance
ARA: Arachidonic acid
BBB: Blood-brain barrier
Ccl: Chemokine (c-c motif) ligand
Cd: Cluster of differentiation
Cox: Cyclooxygenase
cPLA_2_: Cytosolic phospholipase A_2_
DHA: Docosahexaenoic acid
Fcgr: High affinity immunoglobulin gamma Fc region
Gapdh: Glyceraldehyde 3-phosphate dehydrogenase
*Gfap*: Glial fibrillary acidic protein
Hprt: Hypoxanthine-guanine phosphoribosyltransferase
icv: Intracerebroventricular
Ifrd: Interferon-related developmental regulator
Il: Interleukin
Irf: Interferon regulatory factor
LPS: Lipopolysaccharide
Ly: Lymphocyte antigen
Mfsd2a: Major facilitator superfamily domain containing 2A
PUFA: Polyunsaturated fatty acids
qPCR: Quantitative polymerase chain reaction
s.c.: Subcutaneous
SEM: Standard error of the mean
Tnf: Tumor necrosis factor
Trem: Triggering receptor expressed on myeloid cells
