# Supplementary material for: The effects of n-6 polyunsaturated fatty acid deprivation on the inflammatory gene response to lipopolysaccharide in the mouse hippocampus
Source: J Neuroinflammation. 2019 Nov 27;16:237. doi: 10.1186/s12974-019-1615-0 (PMC6882015; doi:10.1186/s12974-019-1615-0)
Supplement: Supplementary file 1 — Additional file 1: Figure S1. Stability of various common reference (housekeeping) genes across diet/surgery groups. Glyceraldehyde 3-phosphate dehydrogenase (Gapdh) and hypoxanthine guanine phosphoribosyltransferase (Hprt). Figures represent n = 5–8 mice per group. Figure S2. Body weights of animals fed n-6 PUFA deprived versus n-6 PUFA adequate before LPS administration and at 1, 3, 7 and 14 days after LPS administration. Bars represent mean ± standard error of the mean of n = 7–8 mice per group. Figure S3. Genes involved in the inflammatory response to LPS administration using a one-way ANOVA (corrected p > 0.05) of the microarray. A) Aif1: allograft inflammatory factor 1, B) Cd86: cluster of differentiation 86 antigen, C) Cd68: cluster of differentiation 68 antigen, D) IL-1β: interleukin 1 beta, E) IL-6ra: interleukin 6 receptor alpha chain, F) Tnf-aip6: tumor necrosis factor alpha induced protein 6, G) Ptgs2 (Cox-2): prostaglandin-endoperoxide synthase 2, and H) Ccl5: chemokine (C-C motif) ligand 5. Bars represent mean ± standard error of the mean of n = 3 mice per group. Significant differences between LPS (day 1 and day 3) and baseline (non-surgery) groups are represented by * (raw p < 0.05). Table S1. List of genes altered by (A) day 1 and (B) day 3 after LPS administration in each diet group. Figures represent n = 3 mice per group. Table S2. List of significantly enriched gene ontology categories in (A) n-6 PUFA adequate and (B) n-6 PUFA deprived at day 3 LPS-administered compared to non-surgery mice. Based on n = 3 mice per group. Benjamini Yekutieli false discovery rate (BY), Gene ontology (GO). [file 12974_2019_1615_MOESM1_ESM.docx]

**SUPPLEMENTARY MATERIAL**

**Additional file 1:**

**Fig. S1** Stability of various common reference (housekeeping) genes across diet/surgery groups. Glyceraldehyde 3-phosphate dehydrogenase (GAPDH) and hypoxanthine guanine phosphoribosyltransferase (hprt). Figures represent n=5-8 mice per group.


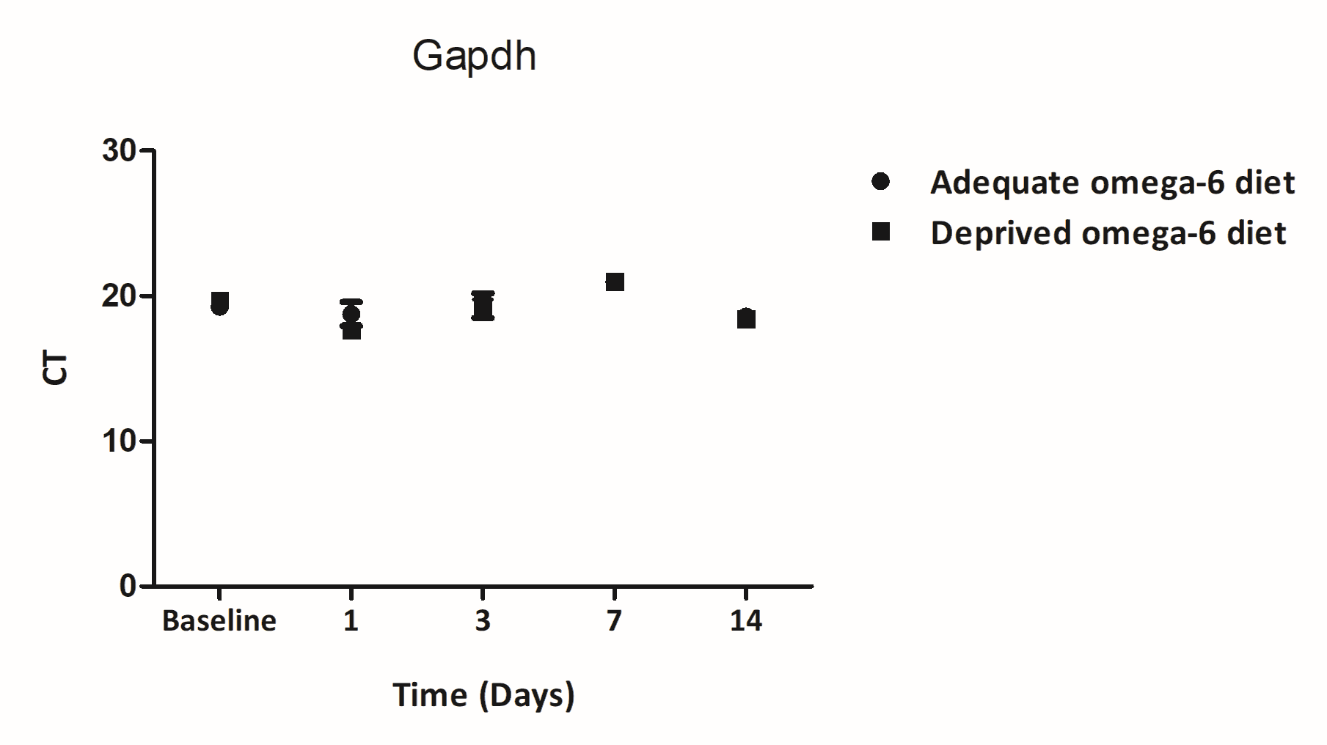


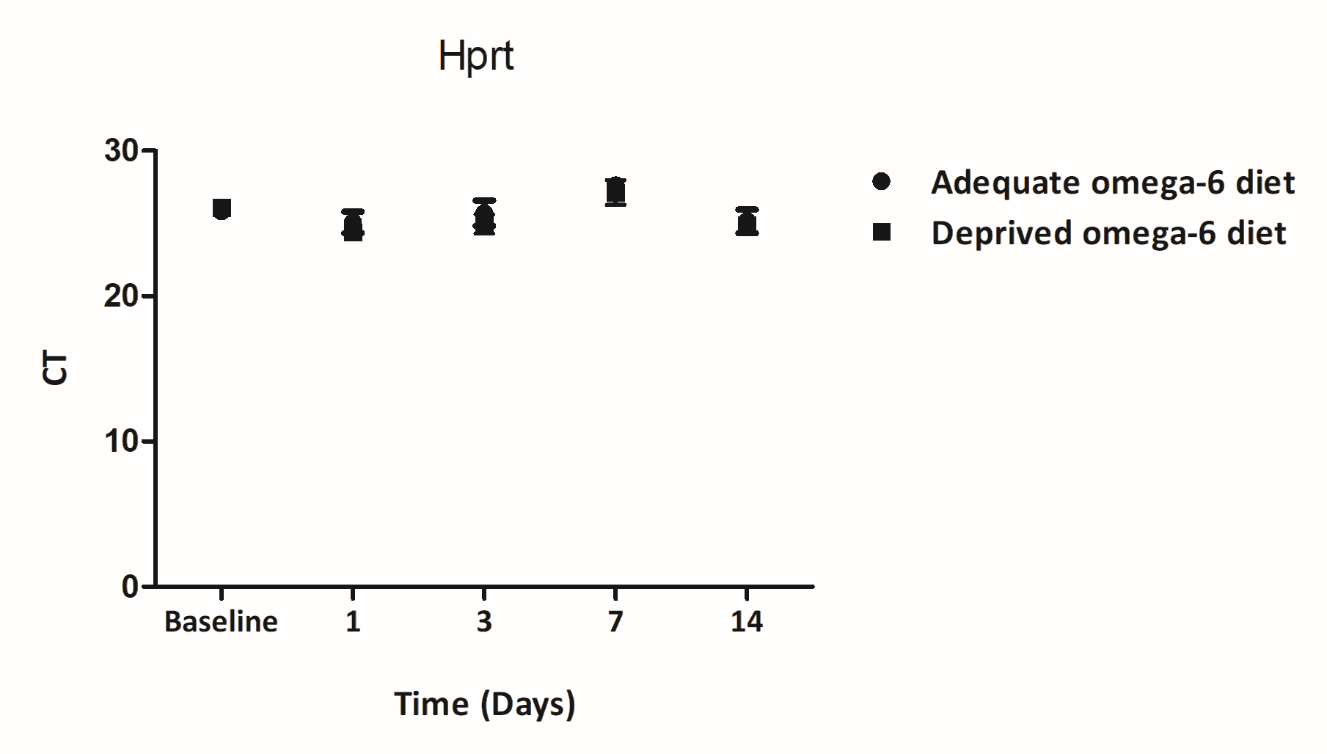


**Fig. S2** Body weights of animals fed n-6 PUFA deprived versus n-6 PUFA adequate diets before LPS administration and at 1, 3, 7 and 14 days after LPS administration. Bars represent mean ± standard error of the mean of n=7-8 mice per group.


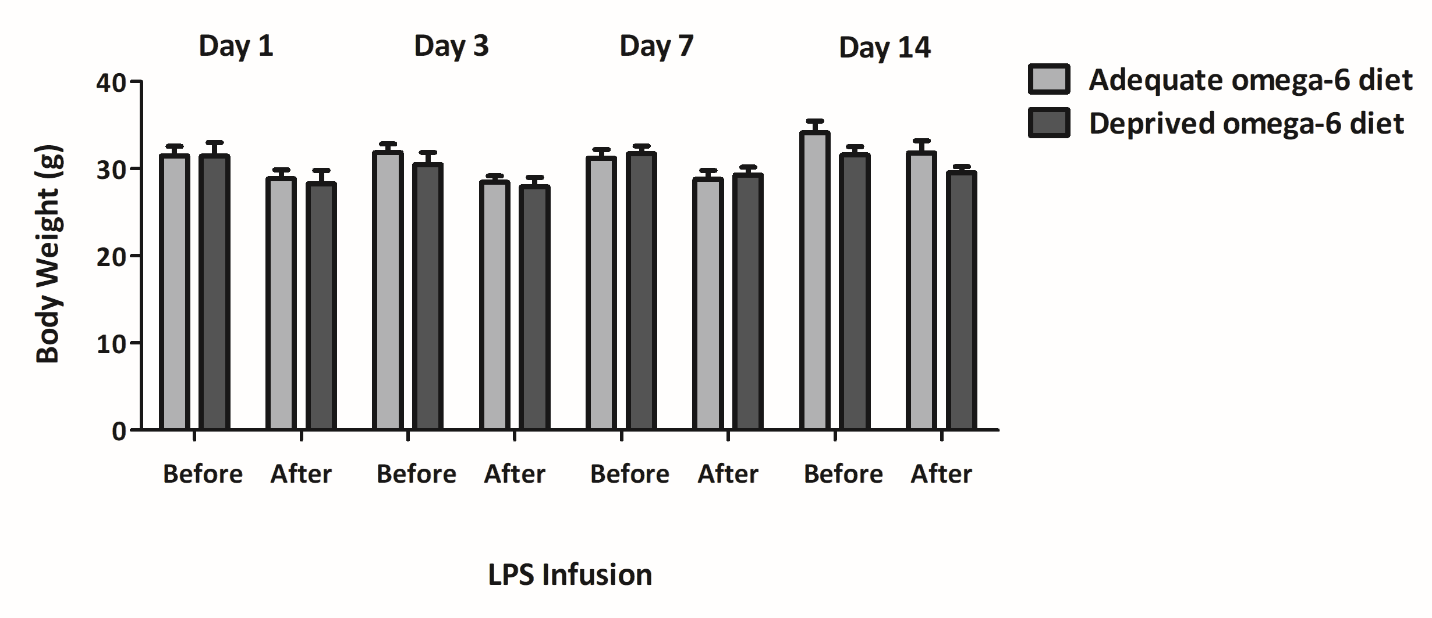


**LPS administration**

**Fig. S3** Genes involved in the inflammatory response to LPS administration using a one-way ANOVA (corrected p>0.05) of the microarray results. A) Aif1: allograft inflammatory factor 1, B) CD86: cluster of differentiation 86 antigen, C) CD68: cluster of differentiation 68 antigen, D) IL-1β: interleukin 1 beta, E) IL-6ra: interleukin 6 receptor alpha chain, F) TNF-aip6: tumor necrosis factor alpha induced protein 6, G) Ptgs2 (COX-2): prostaglandin-endoperoxide synthase 2, and H) Ccl5: chemokine (C-C motif) ligand 5. Bars represent mean ± standard error of the mean of n=3 mice per group. Significant differences between LPS (day 1 and day 3) and baseline (non-surgery) groups are represented by * (raw p<0.05).


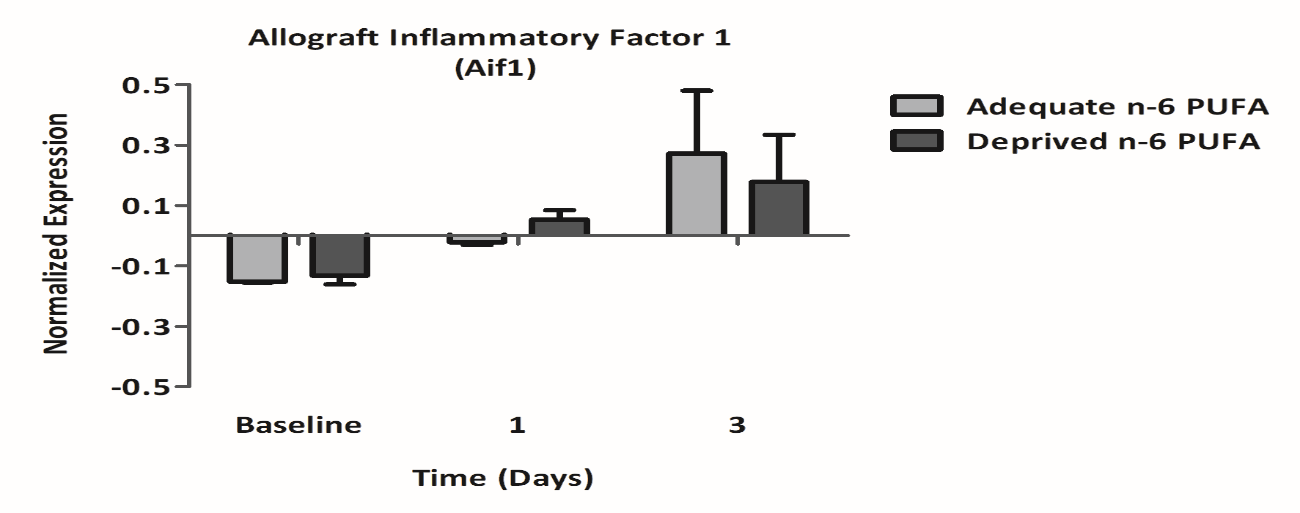
**A**


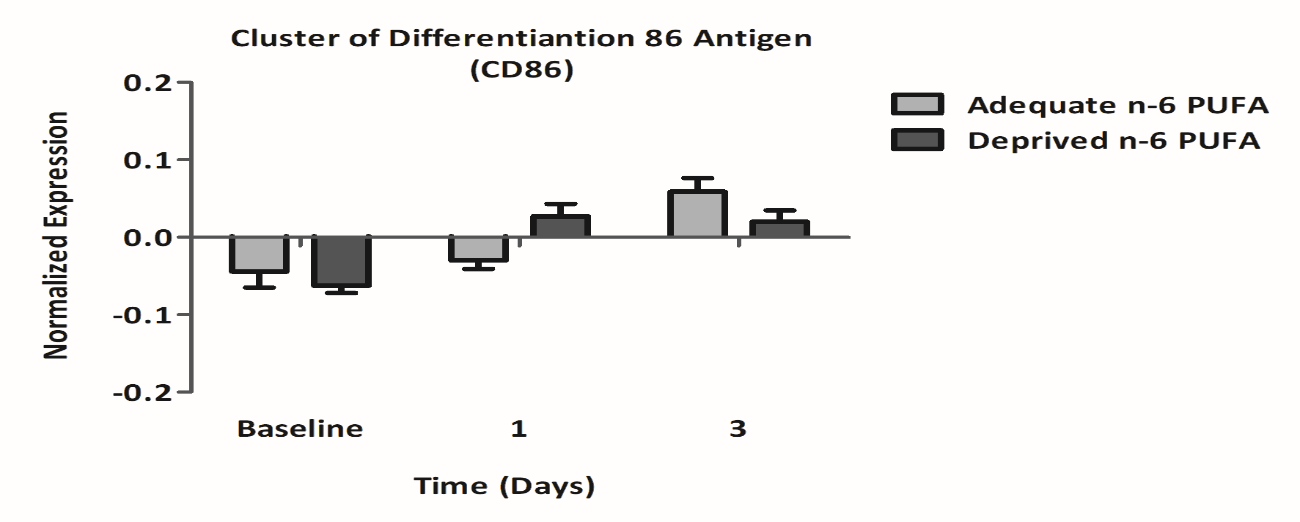
**B**

*

*


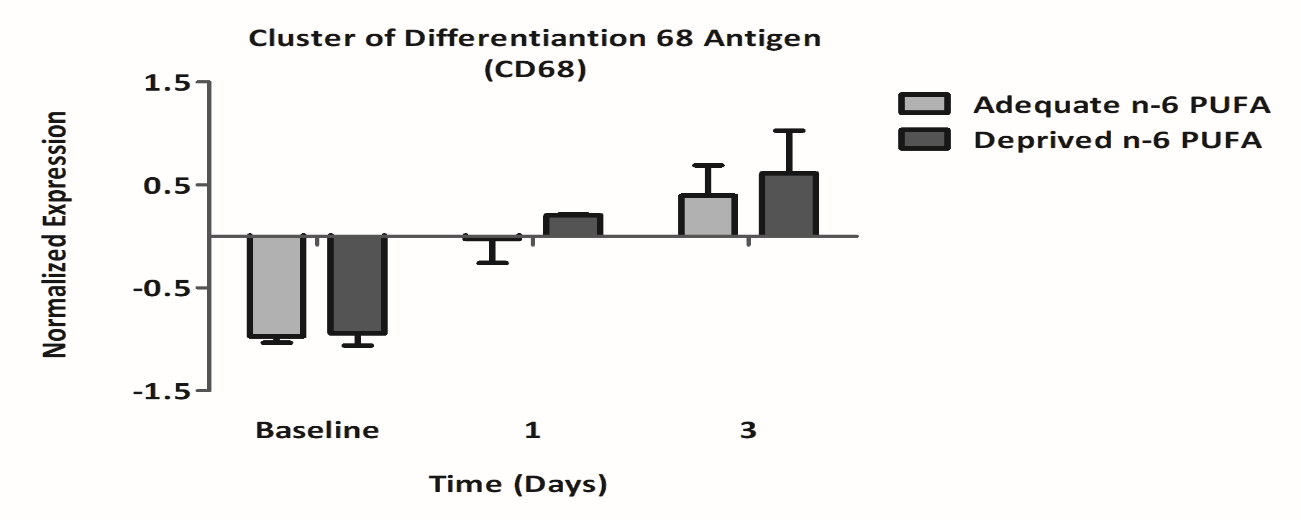
**C**

*

*


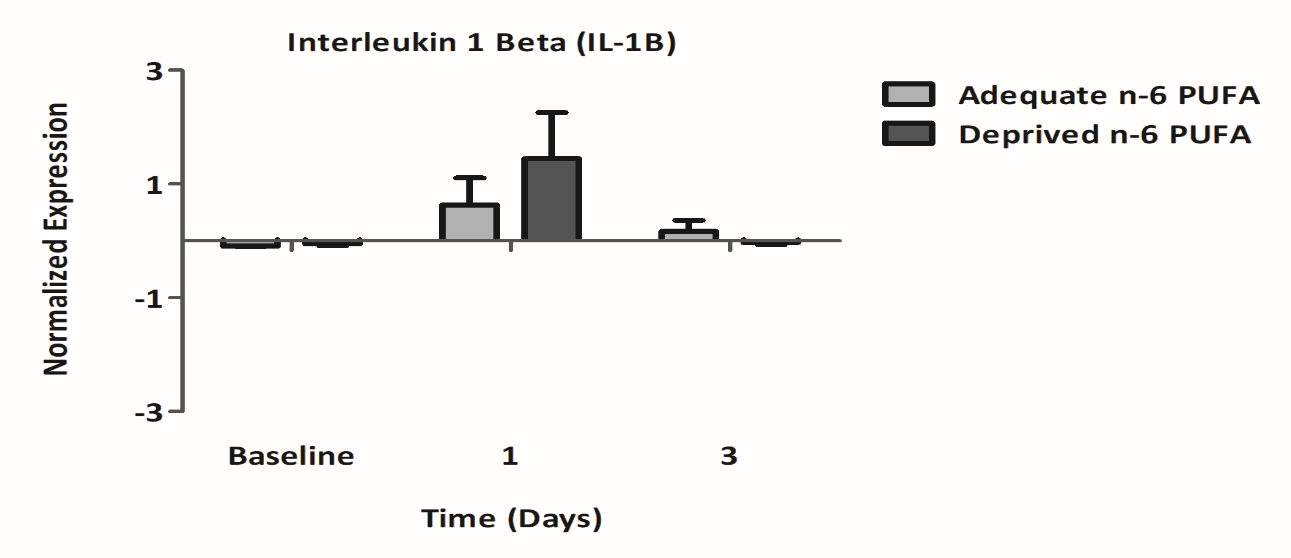
**D**


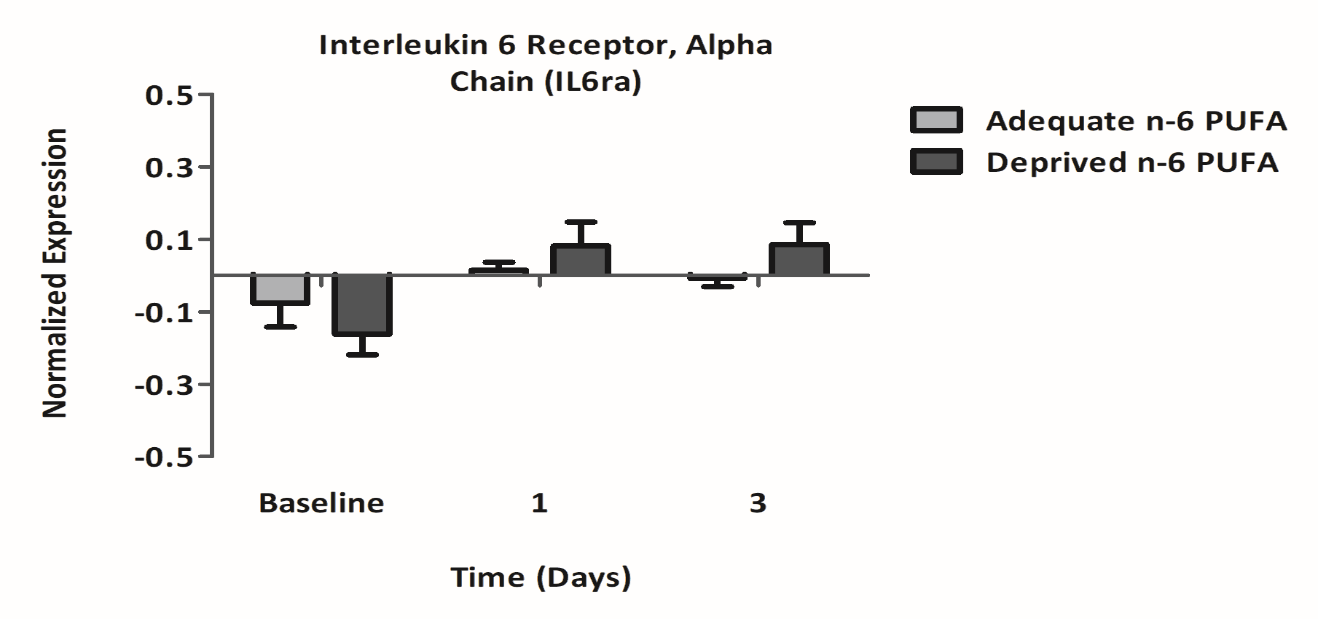
**E**


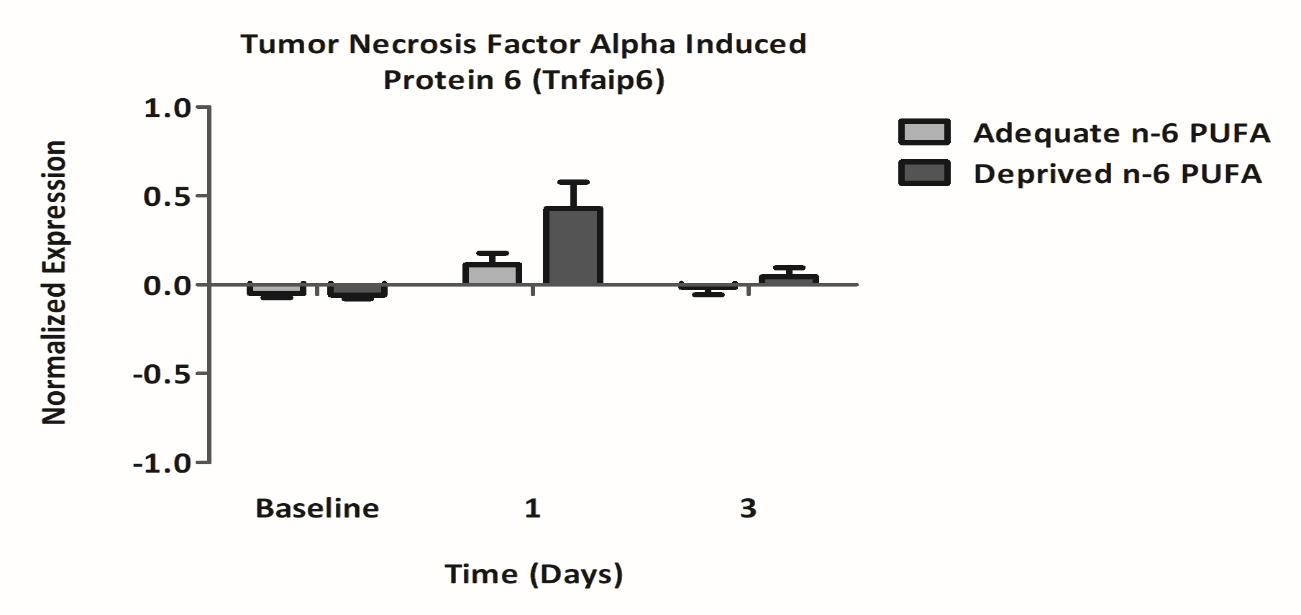


**F**


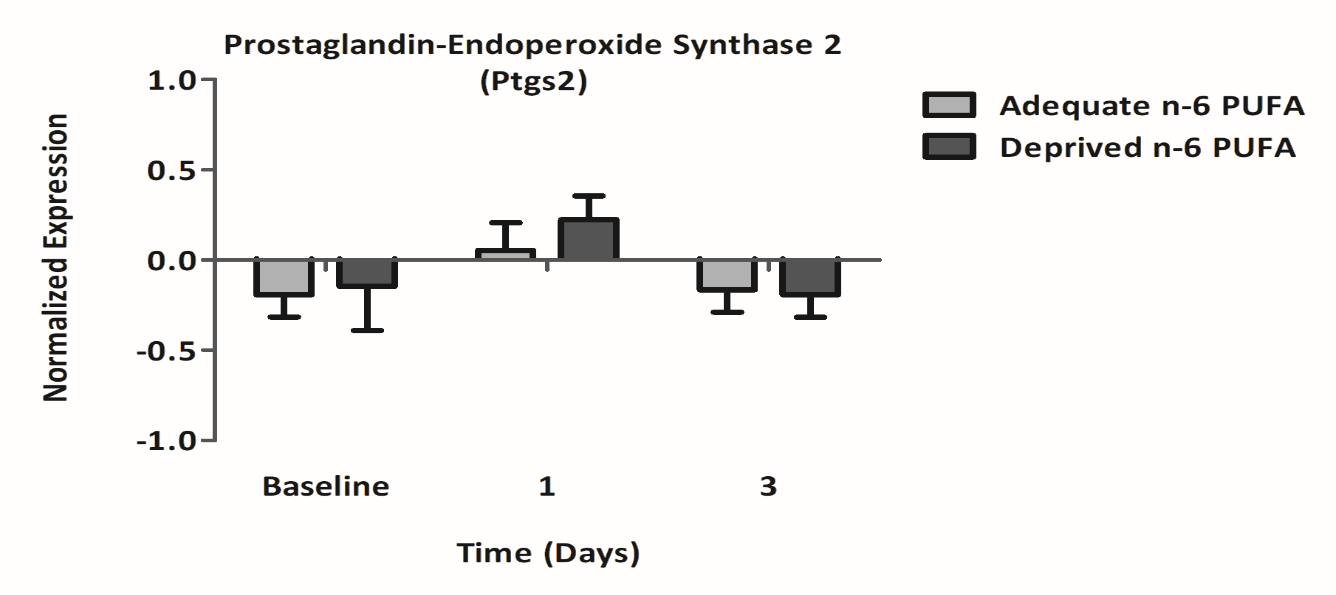
**G**


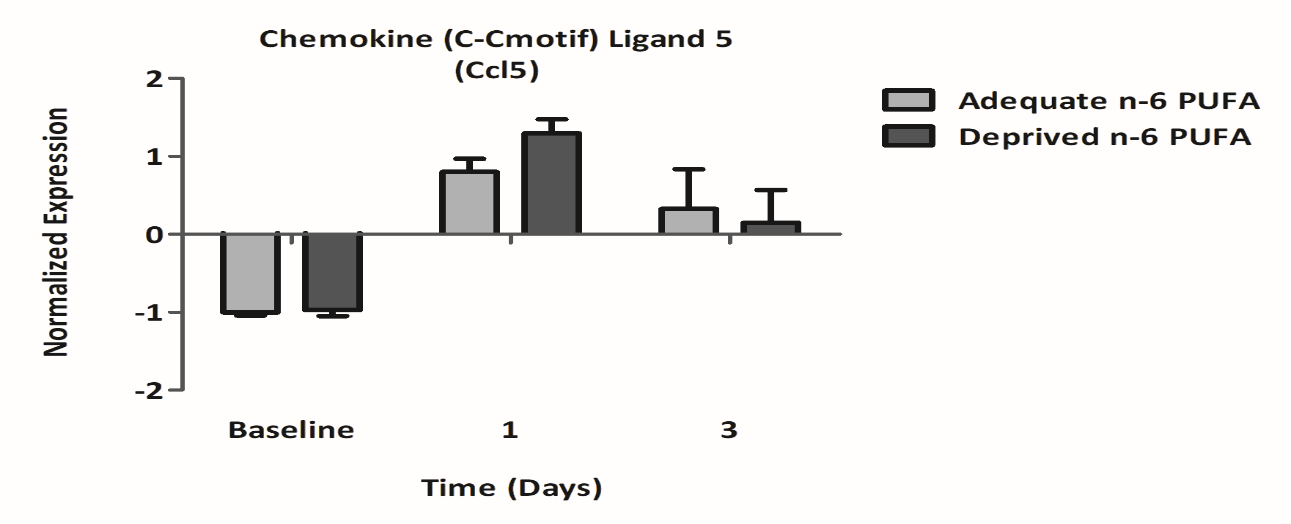
**H**

*

*

**Table S1** List of genes altered by (A) day 1 and (B) day 3 after surgery in each diet group. Figures represent n=3 mice per group.

**A**

| **Genes altered by LPS Administration Shared Between Diet Groups at Day 1** | | |
| --- | --- | --- |
| **Shared between n-6 PUFA deprived and adequate diets** | **Gene Description** | **EntrezGene ID** |
| Stat1 | signal transducer and activator of transcription 1 | 20846 |
| Pla2g4a | phospholipase A2, group IVA (cytosolic, calcium-dependent) | 18783 |
| Srgn | serglycin | 19073 |
| Ccl12 | chemokine (C-Cmotif) ligand12 | 20293 |
| Lgals9 | lectin, galactose binding, soluble 9 | 16859 |
| Gfap\|Gm39395 | glial fibrillary acidic protein\|predicted gene,39395 | 14580//105243489 |
| Lgals3bp | lectin, galactoside-binding, soluble,3 binding protein | 19039 |
| Ly86 | lymphocyte antigen 86 | 17084 |
| Erap1 | endoplasmic reticulum amino peptidase 1 | 80898 |
| Irf9 | interferon regulatory factor 9 | 16391 |
| Ly6a | lymphocyte antigen 6 complex, locus A | 110454 |
| Rtp4 | receptor transporter protein 4 | 67775 |
| Apod | apolipoprotein D | 11815 |
| H2-Q5 | histocompatibility 2, Q region locus 5 | 15016 |
| Slc14a1 | solute carrier family 14 (urea transporter), member 1 | 108052 |
| Ifit1 | Interferon-induced protein with tetra tricopeptide repeats 1 | 15957 |
| B2m | beta-2 microglobulin | 12010 |
| Ctss | cathepsin S | 13040 |
| Fcgr1 | Fc receptor, IgG, high affinity I | 14129 |
| C1qb | complement component 1, q sub component, beta polypeptide | 12260 |
| C1qa | complement component 1, q sub component, alpha polypeptide | 12259 |
| Oasl2 | 2-5 oligoadenylate synthetase-like 2 | 23962 |
| Hadha | hydroxyacyl-Coenzyme Adehydrogenase/3-ketoacyl-Coenzyme Athiolase/enoyl-Coenzyme Ahydratase (trifunctional protein), alpha subunit | 97212 |
| Usp18 | ubiquitin specific peptidase 18 | 24110 |
| Parp12 | poly (ADP-ribose) polymerase family, member 12 | 243771 |
| Pdia4\|Mir704 | protein disulfide isomerase associated 4\|micro RNA 704 | 12304//735289 |
| Hspb6 | heat shock protein, alpha-crystallin-related, B6 | 243912 |
| Ifitm3 | interferon induced transmembrane protein 3 | 66141 |
| Irf7 | interferon regulatory factor 7 | 54123 |
| Psmb10 | proteasome (prosome, macropain) subunit, beta type 10 | 19171 |
| Col6a4 | collagen, type VI, alpha4 | 68553 |
| Magt1 | magnesium transporter 1 | 67075 |

**B**

| **Genes altered by LPS Administration Shared Between Diet Groups at Day 3** | | |
| --- | --- | --- |
| **Shared between n-6 PUFA deprived and adequate diets** | **Gene Description** | **EntrezGene ID** |
| Pla2g4a | phospholipase A2, group IVA (cytosolic, calcium-dependent) | 18783 |
| Fcgr3 | Fc receptor, IgG, low affinity III | 14131 |
| Gfap\|Gm39395 | glial fibrillary acidic protein\|predicted gene, 39395 | 14580///105243489 |
| Lgals3bp | lectin, galactoside-binding, soluble, 3 binding protein | 19039 |
| Npc2 | Niemann-Picktype C2 | 67963 |
| Ly86 | Lymphocyte antigen 86 | 17084 |
| Thbs4 | Thrombospondin 4 | 21828 |
| Hexb | hexosaminidase B | 15212 |
| Ang\|Rnase4 | angiogenin, ribonuclease, RNaseAfamily, 5\|ribonuclease, Rnase A family 4 | 11727///58809 |
| H2-Q5 | histocompatibility 2, Q region locus 5 | 15016 |
| Trem2 | triggering receptor expressed on myeloid cells 2 | 83433 |
| Csf1r | Colony stimulating factor 1 receptor | 12978 |
| Slc14a1 | solute carrier family 14 (urea transporter), member1 | 108052 |
| B2m | beta-2 macroglobulin | 12010 |
| Ctss | cathepsin S | 13040 |
| Fcgr1 | Fc receptor, IgG,high affinity I | 14129 |
| C1qb | complement component 1, q subcomponent, beta polypeptide | 12260 |
| C1qa | complement component 1, q subcomponent, alpha polypeptide | 12259 |
| Hadha | hydroxyacyl-Coenzyme Adehydrogenase/3-ketoacyl-Coenzyme Athiolase/enoyl-Coenzyme A hydratase (trifunctional protein), alpha subunit | 97212 |
| Lair1 | leukocyte-associated Ig-like receptor 1 | 52855 |
| Ifitm3 | interferon induced transmembrane protein 3 | 66141 |
| Col6a4 | collagen, typeVI, alpha 4 | 68553 |

**Table S2** List of significantly enriched gene ontology categories in (A) n-6 PUFA adequate and (B) n-6 PUFA deprived at day 3 LPS-administered compared to non-surgery mice.

**A**

| Significantly enriched GO categories in n-6 PUFA adequate at baseline (non-surgery) vs day 3 LPS-administered mice | | | |  |  |
| --- | --- | --- | --- | --- | --- |
| GO Term | BY Corrected p-value | No. of Genes Driving Enrichment | GO Term | BY Corrected p-value | No. of Genes Driving Enrichment |
| immune response | 3.01E-09 | 12 | negative regulation of cell projection organization | 0.033 | 3 |
| innate immune response | 3.38E-09 | 10 | positive regulation of response to stimulus | 0.033 | 7 |
| immune system process | 1.31E-06 | 12 | regulation of transport | 0.034 | 7 |
| defense response | 3.70E-06 | 10 | response to stimulus | 0.036 | 14 |
| response to stress | 3.27E-04 | 12 | protein dimerization activity | 0.037 | 6 |
| humoral immune response | 5.37E-04 | 5 | extracellular matrix organization | 0.037 | 3 |
| glycoprotein binding | 1.69E-03 | 4 | extracellular structure organization | 0.037 | 3 |
| antibacterial humoral response | 2.79E-03 | 3 | response to external stimulus | 0.039 | 7 |
| antimicrobial humoral response | 3.22E-03 | 3 | regulation of cellular amide metabolic process | 0.040 | 4 |
| positive regulation of biological process | 4.56E-03 | 13 | glycosaminoglycan binding | 0.041 | 3 |
| extracellular space | 5.62E-03 | 8 | lysosome | 0.041 | 4 |
| extracellular region | 7.29E-03 | 12 | anatomical structure development | 0.041 | 11 |
| protein homodimerization activity | 8.84E-03 | 6 | defense response to other organism | 0.041 | 4 |
| antigen processing and presentation of peptide antigen | 0.010 | 3 | lytic vacuole | 0.041 | 4 |
| extracellular region part | 0.012 | 11 | central nervous system development | 0.049 | 5 |
| identical protein binding | 0.013 | 7 | response to organic substance | 0.049 | 8 |
| regulation of endothelial cell proliferation | 0.013 | 3 | cell differentiation | 0.049 | 9 |
| defense response to Gram-positive bacterium | 0.013 | 3 | gliogenesis | 0.049 | 3 |
| response to external biotic stimulus | 0.013 | 6 | multi-organism process | 0.054 | 7 |
| response to other organism | 0.013 | 6 | single-organism developmental process | 0.057 | 11 |
| positive regulation of cell proliferation | 0.013 | 6 | vacuole | 0.057 | 4 |
| system development | 0.013 | 11 | developmental process | 0.057 | 11 |
| response to biotic stimulus | 0.013 | 6 | negative regulation of neuron differentiation | 0.057 | 3 |
| peptide binding | 0.016 | 4 | cellular developmental process | 0.057 | 9 |
| extracellular exosome | 0.016 | 9 | positive regulation of protein secretion | 0.057 | 3 |
| extracellular organelle | 0.016 | 9 | positive regulation of transport | 0.059 | 5 |
| extracellular vesicle | 0.016 | 9 | nervous system development | 0.060 | 7 |
| negative regulation of cell proliferation | 0.018 | 5 | cellular response to lipopolysaccharide | 0.063 | 3 |
| single-multicellular organism process | 0.018 | 12 | negative regulation of cellular component organization | 0.067 | 4 |
| amide binding | 0.018 | 4 | cellular response to molecule of bacterial origin | 0.067 | 3 |
| regulation of smooth muscle cell proliferation | 0.020 | 3 | positive regulation of peptide secretion | 0.071 | 3 |
| regulation of cell proliferation | 0.020 | 7 | positive regulation of molecular function | 0.072 | 6 |
| carbohydrate derivative binding | 0.023 | 8 | regulation of cellular component organization | 0.075 | 7 |
| supramolecular fiber organization | 0.023 | 4 | cellular response to biotic stimulus | 0.077 | 3 |
| response to bacterium | 0.023 | 5 | positive regulation of cellular process | 0.078 | 10 |
| negative regulation of neuron projection development | 0.024 | 3 | glycerolipid metabolic process | 0.079 | 3 |
| antigen processing and presentation | 0.026 | 3 | negative regulation of neurogenesis | 0.087 | 3 |
| multicellular organism development | 0.026 | 11 | regulation of epithelial cell proliferation | 0.088 | 3 |
| Vesicle | 0.028 | 10 | defense response to bacterium | 0.091 | 3 |
| extracellular matrix | 0.031 | 4 | cell body | 0.091 | 4 |
| regulation of localization | 0.032 | 8 | negative regulation of nervous system development | 0.091 | 3 |
| positive regulation of immune system process | 0.033 | 5 | cytokine-mediated signaling pathway | 0.098 | 3 |
|  |  |  |  |  |  |
|  |  |  |  |  |  |
|  |  |  |  |  |  |
|  |  |  |  |  |  |

**B**

| Significantly enriched GO categories in deprived n-6 PUFA at baseline (non-surgery) vs day 3 LPS-administered mice | | | |  |  |
| --- | --- | --- | --- | --- | --- |
| GO Term | BY Corrected p-value | No. of Genes Driving Enrichment | GO Term | BY Corrected p-value | No. of Genes Driving Enrichment |
| immune response | 1.73E-08 | 12 | nervous system development | 0.03 | 8 |
| innate immune response | 1.73E-08 | 10 | positive regulation of secretion by cell | 0.03 | 4 |
| immune system process | 4.91E-07 | 13 | regulation of response to external stimulus | 0.03 | 5 |
| defense response | 1.80E-05 | 10 | negative regulation of cell projection organization | 0.04 | 3 |
| response to stress | 1.95E-04 | 13 | extracellular matrix | 0.04 | 4 |
| humoral immune response | 9.08E-04 | 5 | positive regulation of secretion | 0.04 | 4 |
| antigen processing and presentation of peptide antigen | 9.08E-04 | 4 | regulation of cellular component organization | 0.04 | 8 |
| regulation of transport | 1.03E-03 | 10 | regulation of protein secretion | 0.04 | 4 |
| extracellular region | 1.21E-03 | 14 | regulation of protein transport | 0.04 | 5 |
| regulation of localization | 1.21E-03 | 11 | regulation of leukocyte mediated immunity | 0.04 | 3 |
| positive regulation of immune system process | 1.94E-03 | 7 | extracellular matrix organization | 0.05 | 3 |
| extracellular space | 1.94E-03 | 9 | extracellular structure organization | 0.05 | 3 |
| glycoprotein binding | 1.97E-03 | 4 | regeneration | 0.05 | 3 |
| neuron projection regeneration | 1.97E-03 | 3 | lymphocyte mediated immunity | 0.05 | 3 |
| regulation of immune system process | 2.59E-03 | 8 | protein homodimerization activity | 0.05 | 5 |
| antibacterial humoral response | 2.59E-03 | 3 | single-multicellular organism process | 0.05 | 12 |
| negative regulation of smooth muscle cell proliferation | 2.59E-03 | 3 | immune effector process | 0.05 | 4 |
| antimicrobial humoral response | 3.00E-03 | 3 | regulation of peptide transport | 0.05 | 5 |
| antigen processing and presentation | 3.00E-03 | 4 | glycosaminoglycan binding | 0.05 | 3 |
| response to external biotic stimulus | 3.00E-03 | 7 | regulation of peptide secretion | 0.05 | 4 |
| positive regulation of biological process | 3.00E-03 | 14 | regulation of establishment of protein localization | 0.05 | 5 |
| response to other organism | 3.00E-03 | 7 | regulation of cellular amide metabolic process | 0.05 | 4 |
| positive regulation of response to stimulus | 3.16E-03 | 9 | lytic vacuole | 0.05 | 4 |
| response to biotic stimulus | 3.40E-03 | 7 | adaptive immune response based on somatic recombination of immune receptors built from immunoglobulin superfamily domains | 0.05 | 3 |
| negative regulation of cell proliferation | 3.86E-03 | 6 | lysosome | 0.05 | 4 |
| positive regulation of transport | 4.12E-03 | 7 | cellular component organization | 0.05 | 11 |
| antigen processing and presentation of peptide antigen via MHC class I | 6.07E-03 | 3 | positive regulation of protein kinase activity | 0.05 | 4 |
| response to bacterium | 6.26E-03 | 6 | positive regulation of protein transport | 0.05 | 4 |
| extracellular region part | 6.28E-03 | 12 | endocytosis | 0.05 | 4 |
| regulation of chemokine production | 7.29E-03 | 3 | multicellular organism development | 0.06 | 11 |
| regulation of cell proliferation | 8.19E-03 | 8 | positive regulation of kinase activity | 0.06 | 4 |
| extracellular exosome | 8.19E-03 | 10 | gliogenesis | 0.06 | 3 |
| extracellular organelle | 8.31E-03 | 10 | identical protein binding | 0.06 | 6 |
| extracellular vesicle | 8.31E-03 | 10 | positive regulation of cellular process | 0.06 | 11 |
| positive regulation of immune response | 8.64E-03 | 5 | positive regulation of establishment of protein localization | 0.06 | 4 |
| defense response to other organism | 9.34E-03 | 5 | leukocyte mediated immunity | 0.06 | 3 |
| positive regulation of protein secretion | 0.01 | 4 | cellular component organization or biogenesis | 0.06 | 11 |
| carbohydrate derivative binding | 0.01 | 9 | negative regulation of neuron differentiation | 0.06 | 3 |
| defense response to Gram-positive bacterium | 0.01 | 3 | positive regulation of protein phosphorylation | 0.06 | 5 |
| positive regulation of response to external stimulus | 0.01 | 4 | cell surface | 0.06 | 5 |
| positive regulation of peptide secretion | 0.01 | 4 | regulation of cytokine production | 0.07 | 4 |
| regulation of inflammatory response | 0.01 | 4 | positive regulation of cell proliferation | 0.07 | 5 |
| response to stimulus | 0.01 | 16 | positive regulation of defense response | 0.07 | 3 |
| central nervous system development | 0.01 | 6 | regulation of protein metabolic process | 0.07 | 8 |
| response to external stimulus | 0.01 | 8 | positive regulation of transferase activity | 0.07 | 4 |
| regulation of protein localization | 0.02 | 6 | receptor-mediated endocytosis | 0.07 | 3 |
| Vesicle | 0.02 | 11 | vacuole | 0.07 | 4 |
| peptide binding | 0.02 | 4 | positive regulation of phosphorylation | 0.07 | 5 |
| defense response to bacterium | 0.02 | 4 | regulation of defense response | 0.08 | 4 |
| adaptive immune response | 0.02 | 4 | response to organic substance | 0.08 | 8 |
| amide binding | 0.02 | 4 | neuron projection development | 0.08 | 4 |
| regulation of smooth muscle cell proliferation | 0.02 | 3 | positive regulation of cell communication | 0.08 | 6 |
| regulation of lymphocyte mediated immunity | 0.03 | 3 | positive regulation of signaling | 0.08 | 6 |
| regulation of response to stimulus | 0.03 | 10 | negative regulation of cellular component organization | 0.09 | 4 |
| regulation of immune response | 0.03 | 5 | anatomical structure development | 0.09 | 11 |
| multi-organism process | 0.03 | 8 | cell differentiation | 0.09 | 9 |
| regulation of adaptive immune response based on somatic recombination of immune receptors built from immunoglobulin superfamily domains | 0.03 | 3 | locomotion | 0.09 | 5 |
| immunoglobulin mediated immune response | 0.03 | 3 | glycerolipid metabolic process | 0.09 | 3 |
| cell body | 0.03 | 5 | neurogenesis | 0.09 | 6 |
| system development | 0.03 | 11 | activation of immune response | 0.09 | 3 |
| supramolecular fiber organization | 0.03 | 4 | cellular developmental process | 0.09 | 9 |
| negative regulation of neuron projection development | 0.03 | 3 | negative regulation of neurogenesis | 0.09 | 3 |
| B cell mediated immunity | 0.03 | 3 | multicellular organismal process | 0.09 | 13 |
| regulation of adaptive immune response | 0.03 | 3 | positive regulation of molecular function | 0.09 | 6 |

Based on n=3 mice per group. Benjamini and Yekutieli false discovery rate (BY), Gene ontology (GO).
